# Improving antibacterial ability of Ti-Cu thin films with co-sputtering method

**DOI:** 10.1038/s41598-023-43875-4

**Published:** 2023-10-03

**Authors:** Samaneh Mahmoudi-Qashqay, Mohammad-Reza Zamani-Meymian, Seyed Javad Sadati

**Affiliations:** Department of Physics, Iran University of Science and Technology, P.O. Box 16846-13114, Tehran, Iran

**Keywords:** Biophysics, Microbiology, Chemistry, Materials science, Nanoscience and technology, Physics

## Abstract

Due to the resistance of some bacteria to antibiotics, research in the field of dealing with bacterial infections is necessary. A practical approach utilized in this study involves the preparation of an antibacterial thin film on the surfaces, which can effectively inhibit and reduce biofilm formation and bacterial adherence. In this study, we report the fabrication of bactericidal titanium (Ti) and copper (Cu) surfaces which involves a powerful co-sputtering method. This method provides a situation in which constituent elements are deposited simultaneously to control the composition of the thin film. Prepared samples were examined by energy-dispersive X-ray spectroscopy (EDX), scanning electron microscopy (SEM), X-ray diffraction (XRD), atomic force microscopy (AFM), and contact angle measurements. To evaluate antibacterial behavior, we used two bacterial strains Gram-negative *Escherichia coli* (*E. coli*) and Gram-positive *Staphylococcus aureus* (*S. aureus*). Antibacterial activity of the prepared sample was assessed by determining the number of colony-forming units per milliliter (CFU/ml) using a standard viable cell count assay. Results indicated that as the Cu concentration increased, the nanoscale surfaces became rougher, with roughness values rising from 11.85 to 49.65 nm, and the contact angle increased from 40 to 80 degrees, indicating a hydrophilic character. These factors play a significant role in the antibacterial properties of the surface. The Ti-Cu films displayed superior antibacterial ability, with a 99.9% reduction (equivalent to a 5-log reduction) in bacterial viability after 2 h compared to Ti alone against both bacterial strains. Field emission scanning electron microscopy (FE-SEM) images verified that both *E. coli and S. aureus* cells were physically deformed and damaged the bacterial cell ultrastructure was observed. These findings highlight that adding Cu to Ti can improve the antibacterial ability of the surface while inhibiting bacterial adherence. Therefore, the Ti_14_-Cu_86_ sample with the highest percentage of Cu had the best bactericidal rate. Investigation of toxicity of Cu-Ti thin films was conducted the using the MTT assay, which revealed their biocompatibility and absence of cytotoxicity, further confirming their potential as promising biomaterials for various applications.

## Introduction

Bacterial adhesion and subsequent colonization of the bacteria on surfaces is one of the main reasons for infections in medicine and the food industry^1,2^. Medical instruments, dental implants, orthopedic implants, artificial vessels, food packaging industries, and water purification systems are all exposed to these possible infections^3–8^. It can be said that the most concerning complication of the implantation of biological materials in the human body is infection related to biomaterials^9^. Therefore, prevention of infection and interrupting the mechanism of bacterial adherence to surfaces and removing them is necessary to prevent related problems effectively^10–12^.

Bacterial infections pose a significant threat to human health and cause various diseases. Over the years, antibiotics have played a crucial role in combating bacterial infections by benefiting from technological advancements and medical knowledge^13^. However, excessive and indiscriminate use of antibiotics has resulted in the development of antibiotic resistance among bacterial strains^14^. Antibiotic resistance has become a significant challenge in treating infections, making it increasingly difficult to find effective solutions^15^. To overcome antibiotic resistance, researchers have explored the effectiveness of metal nanoparticles (NPs) and nanocomposite thin films and coatings as potential antibiotics alternatives. These approaches combat bacterial infections through the controlled release of antimicrobial agents and the prevention of bacterial adherence to surfaces^16–19^.

For bacteria to adhere to the surface of a biomedical device, such as an implant, it is necessary for the bacteria to escape from the host implant’s defenses and adhere to it to form a microcolony and, finally a biofilm, which is a three-dimensional bacterial community^20,21^. Since destroying bacteria after biofilm formation is more challenging, preventing bacterial adhesion and creating biofilm is a more effective solution^22^. Various factors such as physicochemical properties, environmental conditions, and surface morphology effectively control biofilm formation^23–25^.

Undoubtedly, hindering the adhesion of bacteria to the surface and preventing biofilm formation is an essential challenge for researchers in developing antibacterial characters and their fabrication methods, which require intricate physical and chemical mechanisms^26–28^. In addition to using antibacterial compounds, designing and modifying surface properties to prevent biofilm formation is another effective strategy to overcome surface-adherent bacterial contamination^29–31^. Surface properties such as roughness^32,33^, wettability^34^, pattern^35^, and surface energy^36^, play essential roles in bacterial adhesion.

The effect impact of surface roughness, an important surface characteristic, on bacterial adhesion differs depending on the roughness scale^37^. At the nanoscale level, surfaces with roughness exhibit the best anti-adhesion properties, whereas at the micro-scale level, rougher surfaces tend to promote bacterial adhesion^38^. The difference in bacterial adhesion is due to the contact points to which the bacteria can adhere^39^. Generally, bacteria prefer smoother surfaces at a nanometer scale that provide them with an opportunity to grow and produce a significant amount of extracellular polymeric substance (EPS), which aids in their survival^40,41^. Lüdecke et al*.* showed that a slight change in the roughness parameter (RMS) of the TiO_2_ coating can decrease the adherence of *Escherichia coli* (*E. coli*)^42^. Moreover, other research reports have suggested that surface roughness can control colony forming of the bacteria on Ti-based dental implants^43^. Jang et al*.* investigated the effect of surface roughness on bacterial adhesion to nitinol (NiTi) wire surfaces. Their results demonstrated a significant reduction in bacterial adhesion by polishing the surfaces to a nano-level roughness^44^. Petrini et al. examined the surface characteristics of two Ti_6_Al_4_V surfaces produced by selective laser melting (SLM) and treated with electrochemical (EL) polishing, and organic acid etching (OEA). The OEA samples showed higher nano roughness, improved wettability, and reduced bacterial biofilm formation compared to the machined and EL samples^45^. However, investigating the surface roughness and its effect on the antibacterial mechanism in the nanoscale presents numerous challenges and questions that need to be clarified as a new research scope.

Additionally, wetting is considered another influential factor in the adhesion of bacteria on surfaces, as it can either prevent colony growth or accelerate it^46^. Surface wettability is typically obtained by contact angle measurement techniques, which usually show that a higher level of bioactivity is associated with a lower contact angle of the hydrophilic surfaces. This is an essential factor in determining the tissue compatibility of biomaterials^47^. Of course, the issue of bacterial adherence on hydrophilic surfaces is controversial and ambiguous. Some studies have reported anti-adhesion properties for hydrophilic surfaces, while others have reported cell adhesion on such surfaces^48–50^.

An ideal antibacterial surface exhibits two main properties: prevention of bacterial adherence, and bacterial elimination^51^. In addition, biocompatibility, non-toxicity, accessibility, and cost-effectiveness are other essential considerations for the fabrication, modification, and use of antibacterial surfaces and coatings^52^.

Titanium (Ti) is widely utilized in the biomedical industry owing to its antibacterial properties, significant corrosion resistance, and mechanical properties^53^. Moreover, these promising properties, the addition of metals such as aluminum (Al), copper (Cu), gold (Au), and silver (Ag) improve and enhance biocompatibility of Ti. Cu to its distinct chemical and physical properties can hold excellent promise as an antibacterial material, alongside Ti^54^. However, according to the significant advancements in understanding the antibacterial potential of different Ti alloys, it is imperative to conduct further research to comprehend their surface properties. So, there is a need for a comprehensive investigation into the effect of Cu, and its concentration impact on the nanoscale roughness and wettability parameters, which remains an unexplored subject in the field of antibacterial properties.

Recently, various approaches such as spray deposition, spin-coating, sol–gel, acid etching, anodic oxidation, physical vapor deposition (PVD), and electrodeposition have been employed to modify antibacterial surfaces, and alter their surface properties, and topology^55^. Among these methods, magnetron sputtering has been shown in several studies to be a suitable and feasible way to modify antibacterial coatings^56–58^. This method offers an excellent opportunity to produce homogeneous, smooth, and dense coatings with a rapid deposition rate, thereby increasing the coating-substrate adherence^59,60^. Additionally, multicomponent composite layers can be formed with precise control of the component concentrations by the magnetron co-sputtering^61–65^. For instance, previous research has demonstrated the successful use of TiN radio frequency sputtering in the coating of Ti-25Ta-xZr nanotube alloys for use in dental implants^66^. In addition, titanium boron nitride thin films suitable for use as protective coatings for metallic implants were fabricated using pulsed DC magnetron sputtering^67^. Magnetron sputtering was used to deposit Ti-Me intermetallic (Me = Al, Cu, Ag, Au) thin films, which were then characterized to assess their potential for use as biopotential electrodes in noninvasive physiological monitoring^68^. These examples highlight the broad range of biomedical devices that can benefit from this coating technique to, biocompatibility, durability, and functionality.

This paper presents the preparation of Ti–Cu films involving different ratios of Cu to Ti, and examines their antibacterial performance, which shows excellent antibacterial properties. Magnetron co-sputtering method was employed to fabricate these films. The primary objective of this study was to establish a significant relationship between the percentage of constituent elements and surface characteristics, such as the surface roughness and wettability of Ti-Cu thin films. By assessing the impact of Ti-Cu thin films on bacterial adhesion and gaining a clearer understanding of their antibacterial properties, we aimed to contribute to the knowledge in this field.

## Experimental

### Thin film preparation

#### Substrate treatment

First, the glass slides (Medical Lab Unground Edges Microscopy Glass Slide 7102) were prepared as substrates. They were cut in dimensions of 1 × 1cm. Then, they were sonicated in deionized water, ethanol, and acetone for 15 min, respectively, and subsequently dried by a thermal dryer. The silicon (Si) substrate was also prepared using the same method and utilized for specific characterization analyses.

#### Deposition condition

**Figure 1:**
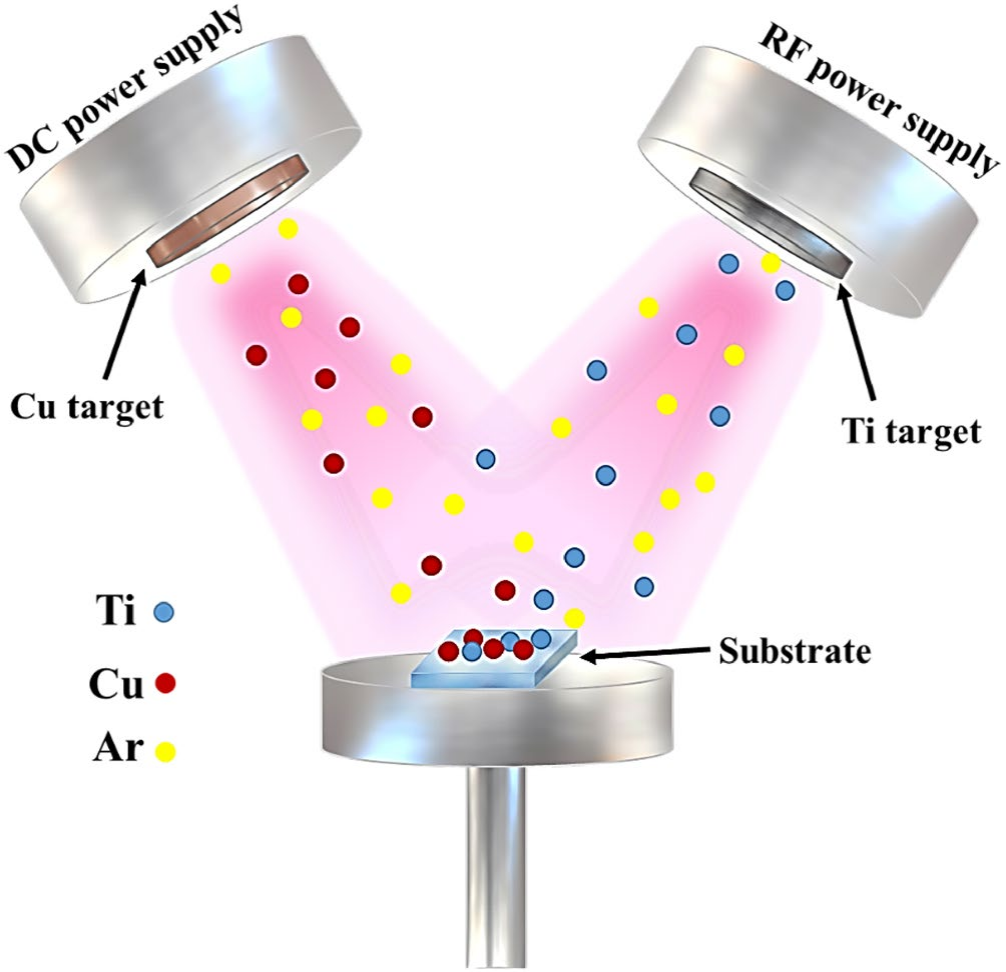
Schematic images of the preparation of Ti-Cu thin films using the co-sputtering method.

The Ti-Cu thin films were deposited on glass and Si substrates using the co-sputtering method with a Desk Sputter Coater DST3-A (Nanostructure Coating Co.). Radiofrequency (RF) magnetron sputtering was used for the Ti target (99.9 at. % purity), while direct current (DC) magnetron sputtering was employed for the Cu target (99.9 at. % purity). The schematic representation of Ti-Cu thin film preparation through the co-sputtering method is shown in Fig. 1. Before film deposition, both metal targets were pre-sputtered for 5 min to remove contaminations and oxide layers. The base pressure was 9 × 10^–5^ Torr, and the argon (Ar) flow rate was 33 sccm (standard cubic centimeter per minute). The Ti-Cu thin films with various elemental percentages were named as follows: Ti, Ti_81_-Cu_19_, Ti_49_-Cu_51_, Ti_29_-Cu_71_, and Ti_14_-Cu_86_, respectively. Table 1 summarizes the voltage and current applied to the Cu target and other deposition parameters used to prepare Ti-Cu thin films.

**Table 1:** Magnetron sputtering process condition.

Samples	RF power, [W]	DC current, [mA]	DC voltage, [V]	Deposition time [s]	Working pressure [Torr]	Substrate rotation speed [rpm]
Ti	150	0	0	1020	2 × 10^–2^	18
Ti_81_-Cu_19_	150	10	249	1020	2 × 10^–2^	18
Ti_49_-Cu_51_	150	50	319	1020	2 × 10^–2^	18
Ti_29_-Cu_71_	150	60	325	1020	2 × 10^–2^	18
Ti_14_-Cu_86_	150	90	362	1020	2 × 10^–2^	18

### Characterization

#### Interfacial analysis of prepared thin films

The elemental compositions of prepared thin films on Si substrates were examined using energy dispersive X-ray (EDX) spectroscopy with field emission scanning electron microscopy (FESEM) from Zeiss Co. (Germany), and MIRA3 TESCAN, respectively. Five different elemental measurements and EDX maps were taken for each thin film, and the average values were reported. The thickness of the thin films was also investigated using scanning electron microscopy (SEM) from TESCAN VEGA//XMU. The structural properties of the deposited coatings were determined using X-ray diffraction (XRD) with an X' Pert Pro instrument from Panalytical Co. (UK) and Cu Kα radiation (λ = 0.15406). The Grazing incidence XRD (GIXRD) technique was employed with a low grazing angle of 1°. The topography and roughness of the surface were evaluated with the atomic force microscopy (AFM) by the ARA-AFM model, Ara Research Co. A sample area of 5 × 5 μm^2^ was scanned in the non-contact mode. The contact angle method was used to determine the wettability of the surface. Water drops were placed on the thin film samples, and the image of the drop was taken by the camera. The contact angle of the samples was measured with a digital light microscopy DINOLITE-AM model 4113ZT made in Taiwan and a 10 µL Hamilton syringe (USA). Typically, droplets with volumes ranging from 1 to 10 µL are employed for contact angle measurements.

### Antibacterial test

#### Colony-forming units’ assay

The antibacterial properties of Ti-Cu thin films with varying concentrations of Cu against *S. aureus ATCC 6538*, and *E. coli ATCC 25,922* were evaluated using the colony-forming units (CFU/ml) assay. The direct contact method was used to determine the viable cell count. The experiment was conducted at four different time points: 30 min, 2 h, 6 h, and 24h. Trypticase Soy Agar (TSA) was used as the solid culture medium, while Muller Hinton Broth (MHB) was used as the liquid culture medium. The bacterial suspension was prepared with a concentration of 1 × 10^6^ CFU/ml in a dilution solution of phosphate buffer. A volume of 1.0 ml of the suspension was placed on the sample surfaces and were incubated at 35°C ± 2 °C. Then, 0.1 ml of the suspension was taken in 30 min, 2 h, 6 h, and 24 h and cultured on TSA agar using the plate spread method. The plates were then incubated at 35°C ± 2 °C for 48 h. The humidity level was set at 90% throughout the experiment, providing an optimal environment for bacterial growth. The number of colonies formed on the plates was counted using a colony counter. The CFU/ml was calculated using the following equation:

1 $${\text{CFU}}/{\text{ml }} = \, \left( {{\text{number of colonies }} \times {\text{ dilution factor}}} \right)/{\text{volume of culture plate}}.$$

The antibacterial activity can be determined using the below formula:

2 $${\text{Antibacterial activity }} = {\text{ Log }}\left( {{\text{A}}/{\text{B}}} \right).$$

In this formula, a represents the number of CFU/ml on the surface of the reference sample, while B represents the number of CFU/ml on the surface of the treated sample.

The percentage of bacterial and logarithmic reduction were calculated based on the CFU/ml. This work follows National Standard No. 10900, derived from ISO 22196:2011, and provided a method for measuring the antibacterial activity of plastics and other non-porous surfaces^69^.

#### FESEM analysis

FESEM images can be crucial in investigating and identifying antibacterial agents, by providing high-quality images. These images help determine the details of bacterial variations, such as the state of the cell membrane, structure, and distribution of bacteria in response to antibacterial agents. The Ti_49_-Cu_51_ and Ti_14_-Cu_86_ thin films, which are antibacterial substances were exposed to *E. coli* and *S. aureus* bacteria, as described in the previous section. First, the bacterial suspension is carefully dropped onto a filter membrane, such as a polycarbonate “nuclepore” filter with a pore size of 0.45 µm or 0.2 µm. The droplets are then allowed to air dry. The dried bacterial samples on the filter membrane are fixed in 2.5% glutaraldehyde in PBS buffer for 45 min, followed by an additional 1 h fixation period. After fixation, the samples are rinsed for 15 min in PBS buffer to remove any residual fixative. The samples are further fixed by immersing them in 1% OsO4 in PBS buffer for 1 h to enhance contrast and preserve the cellular structure. For dehydration, the samples undergo a series of ethanol rinses, 10 min each in 30%, 50%, 70%, 80%, and 100% ethanol. These rinses progressively remove water from the samples. The dehydrated samples are then immersed in absolute ethanol for approximately 15 min to ensure complete water removal. Critical-point drying was performed to transition the samples from the liquid to the gas phase while preserving their structure. The samples were placed in a critical point drying apparatus, where the chamber pressure and temperature were adjusted to reach the critical point of a suitable liquid, such as carbon dioxide (CO_2_). To ensure a gentle drying process without causing sample distortion or damage. To prevent the accumulation of electric charge on the surface, the dehydrated and dried samples were covered with a thin film, approximately 20 nm, conductive layer of gold. The gold coating was deposited using a suitable sputter coater system model DSR1. The exact sputtering parameters, such as the sputtering time, power, and pressure, are 300 s, 27%, and 90 $\times {10}^{-3}$ Torr, respectively. Finally, the prepared samples are ready for FE-SEM analysis. The samples were loaded into a FESEM (MIRA3 TESCAN(, and imaged at high resolution to investigate the bacterial morphology and other sample features.

### Toxicity testing method

The cytotoxicity of the samples was evaluated using the 3-(4,5-dimethylthiazol-2-yl)-2,5-diphenyltetrazolium bromide (MTT) assay to assess their biocompatibility. This commonly used method measures the viability of cells by converting MTT dye into purple formazan crystals within the mitochondria. The concentration of the dye is proportional to the number of viable cells, allowing for quantification with a photometer. The methodology and evaluation criteria of this assay adhere to the ISO standard 2009:5-10993^70^.

In this study, we tested samples Ti_81_-Cu_19_ and Ti_14_-Cu_86_, representing the lowest and highest percentages of Cu, respectively. The samples were sterilized using the UV method before were examined. Control samples (wells with no treatment) and wells containing samples Ti_81_-Cu_19_ and Ti_14_-Cu_86_ were used to establish the baseline cytotoxicity level using the MTT assay. To perform the assay, 1 × 10^4^ L929 cell samples were seeded in 12-well plates with DMEM culture medium supplemented with 10% fetal bovine serum (FBS), 1% penicillin, and streptomycin. This experimental setup was performed thrice. Subsequently, the samples Ti81-Cu19 and Ti14-Cu86 were added to the respective wells, also in triplicate. The plates were then incubated at 37 °C with 5% carbon dioxide for 24 ± 2 h to allow cell-surface interaction. At specified intervals, 100 µL of MTT dye with a 5 mg/ml concentration was added to each well. After 3 h, the purple-colored crystals were dissolved using dimethyl sulfoxide (DMSO). The amount of dissolved color in the DMSO solution was measured with an enzyme-linked immunosorbent assay (ELISA) reader. Higher optical density (OD) values indicate wells with viable cells compared to wells with dead cells. By comparing the optical density of the test samples to the control samples using the following formula:

3 $$\mathrm{\%\,\, Cell \,\,Viability}=\frac{\mathrm{mean\,\, of \,\,OD \,\,sample}}{\mathrm{mean\,\, of\,\, OD\,\, control }}.$$

By employing this formula, the relative viability of the test samples can be determined compared to the control sample and we can provide insights into their cytotoxicity.

### Statistical analysis

We repeated the magnetron sputtering coating process multiple times for each percentage and created several thin layers. Every thin film was tested at least thrice under the same conditions for EDX, antibacterial, and MTT tests. To analyze the obtained data and determine the significant differences among the groups, we employed Duncan’s multiple range test (P < 0.05). This statistical method allowed for the comparison of means and identification of significant variations. Statistical analysis of each measurement was calculated using Origin software and it’s presented in Tables 1S–3S in the Supplementary file.

## Results

### Composition analysis

**Figure 2:**
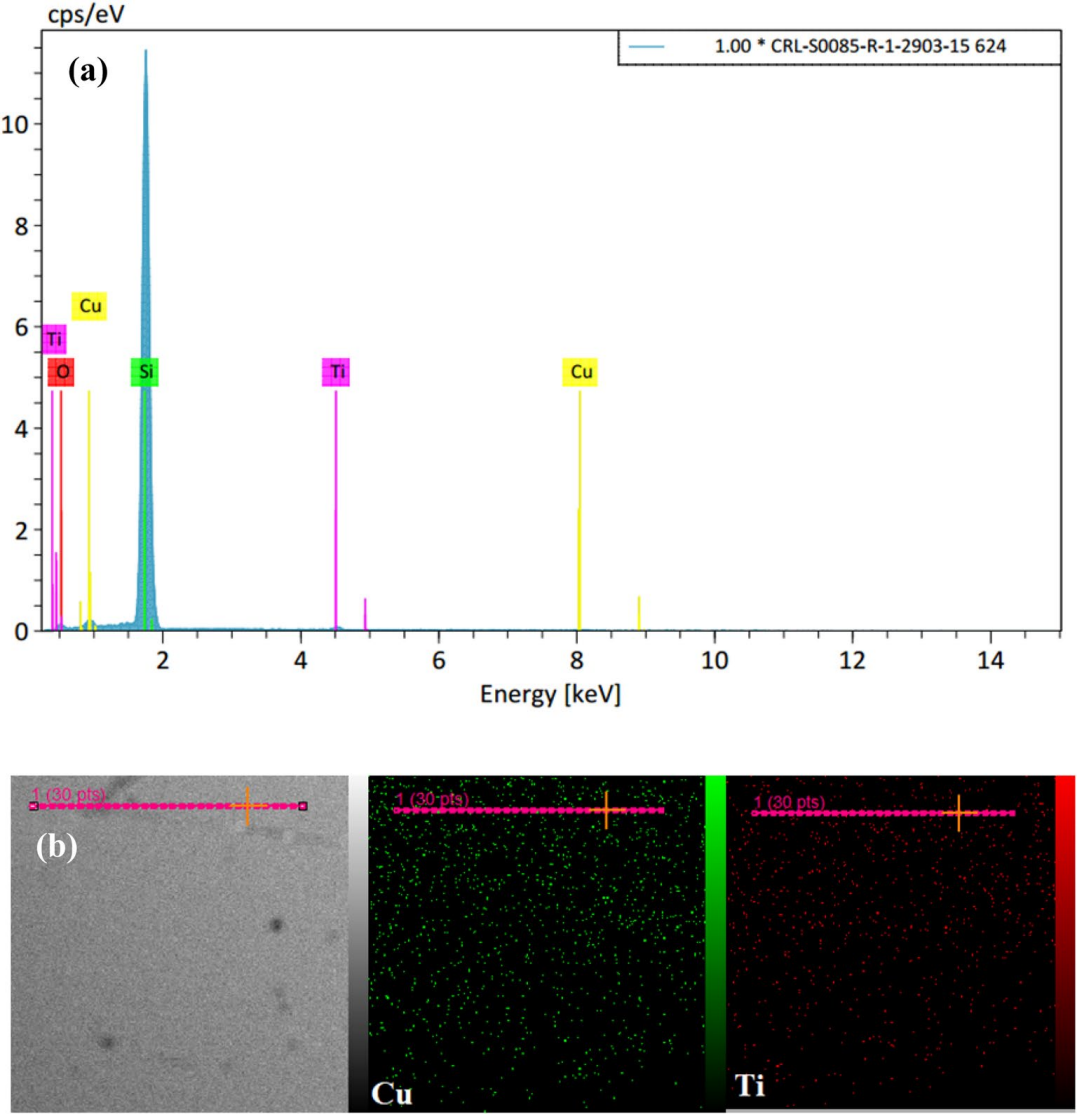
(**a**) EDX spectra and (**b**) Elemental mapping of the sample Ti_49_-Cu_51_.

**Figure 3:**
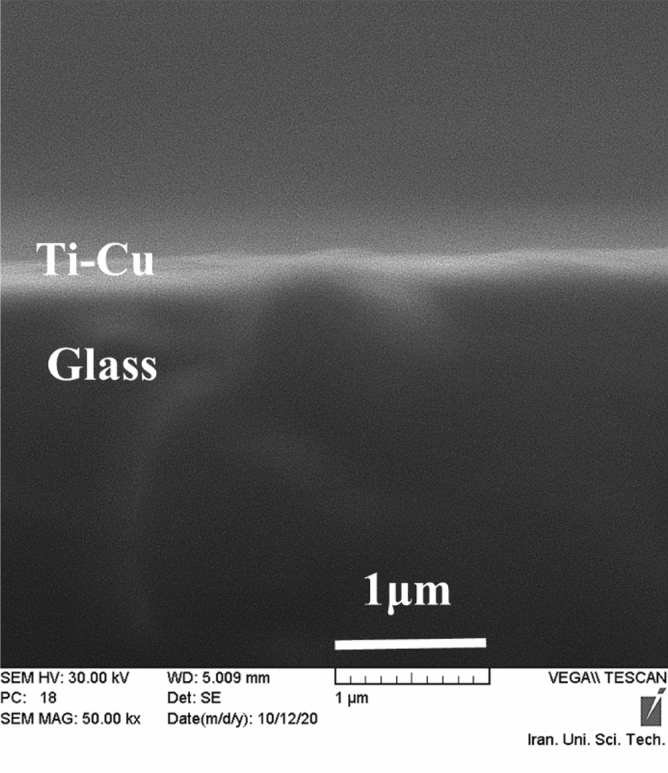
The SEM image of the cross-section view of the sample Ti_49_-Cu_51_.

**Figure 4:**
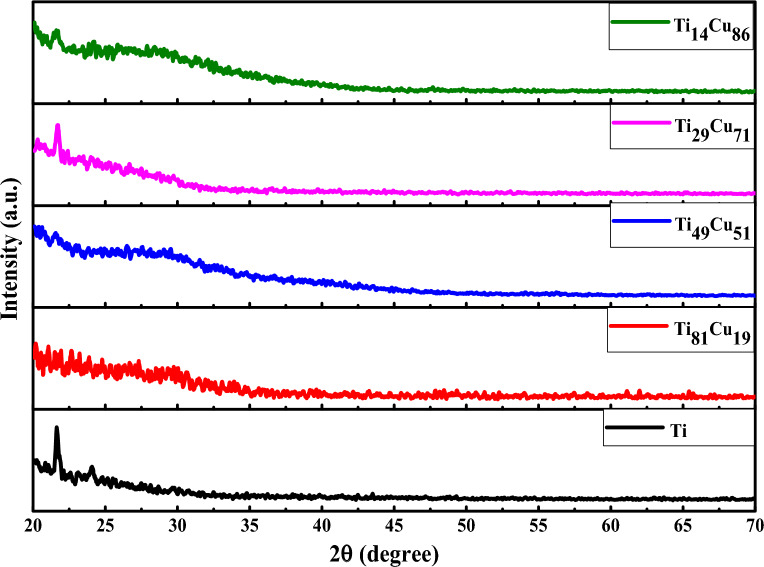
GIXRD pattern of the Ti-Cu thin films on the glass substrate.

The elemental analysis of samples involved the evaluation Ti and Cu values using the EDX technique. The analysis was conducted on all thin films coated by co-sputtering on a pure Si substrate. For instance, the EDX spectrum of the Ti_49_-Cu_51_ thin film is shown in Fig. 2a, while the remaining EDX spectra can be found in Figs. 1S–4S. The percentage of atom and mass of each samples were listed in Table 2. The peaks for Si and O are related to the elements present in the Si substrate. Similarly, the peaks observed for Ti and Cu confirm the presence of these elements and provide their quantities in all samples. To ensure the homogeneous distribution and absence of agglomeration of Ti and Cu particles, the analysis was repeated on 5 points on all sample surfaces. For example, the mapping spectrum of the Ti_49_-Cu_51_ thin film can be found in Fig. 2b, which confirms the uniform distribution of Ti and Cu elements in the prepared samples and agrees with the EDX results. Additionally, Fig. 3. presents a cross-section SEM image of the Ti_49_-Cu_51_ thin film sample, providing an estimate of its thickness, which ranges from 160 to 200nm. The raw data of the SEM image is given in Fig. 5S of the Supplementary file.

**Table 2:** Mass and atomic percentages of elements in each thin film.

Samples	Element			
	Ti	Cu	Ti	Cu
	% Mass		% Atom	
Ti	100	0.00	100	0.00
Ti_81_-Cu_19_	80.78	19.21	84.80	15.20
Ti_49_-Cu_51_	48.85	51.15	55.81	44.18
Ti_29_-Cu_71_	28.74	71.25	28.57	71.42
Ti_14_-Cu_86_	14.03	85.97	17.74	82.25

### Structure analysis

The Grazing X-ray diffraction spectra of the deposited Ti-Cu thin films on a glass substrate with different ratios of Cu to Ti are demonstrated in Fig. 4. It was determined that all binary Ti-Cu thin films had amorphous structures, as there were no Bragg diffraction peaks visible. The peaks at around 2ϴ = 21° could be related to the substrate^71^. The raw data of the GIXRD is given in Fig. 6S of the Supplementary file.

### Antibacterial activities assay of Ti-Cu thin films

**Figure 5:**
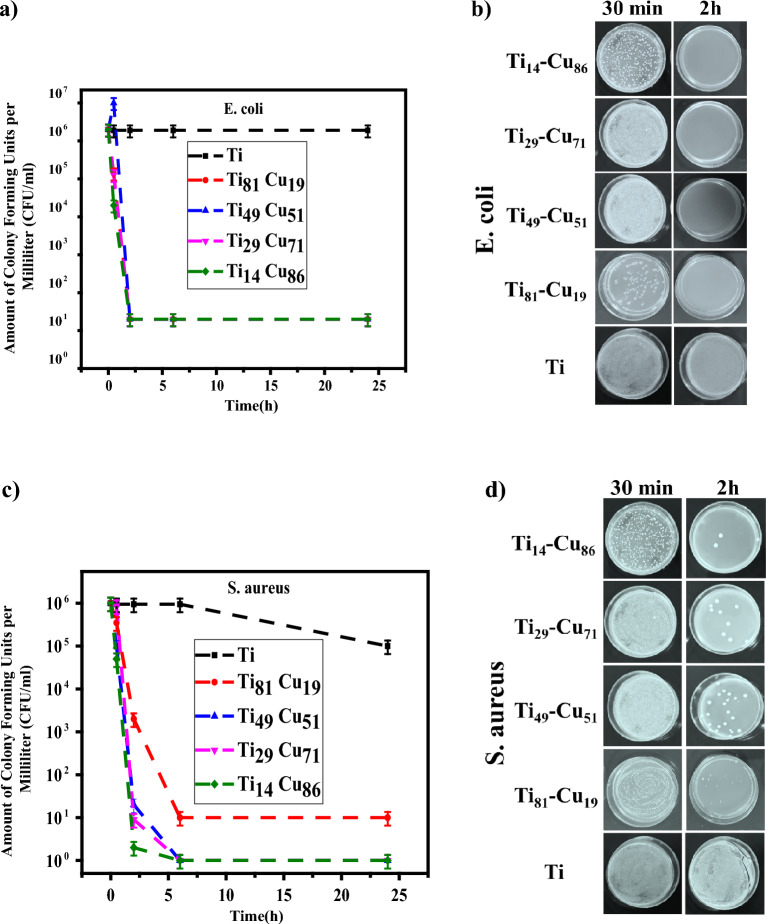
(**a**) CFU/ml analysis graph for *E. coli* bacteria (**b**) Photographic images of *E. coli* antibacterial agar plate counting test in 30 min and 2 h (**c**) CFU/ml analysis graph for *S. aureus* bacteria (**d**) Photographic images of *S. aureus* antibacterial agar plate counting test in 30 min and 2 h. The data shown are representative of (n = 3) repeats.

The antibacterial properties of Ti-Cu thin films were thoroughly investigated using the CFU/ml analysis. The aim of this analysis to examine the survival of the Gram-positive Bacterial *S. aureus* and the Gram-negative Bacterial *E. coli* at varying exposure times, as illustrated in Fig. 5a–d. In this method, the percentage of bacteria reduction is computed by comparing the number of bacteria before (n_i_) and after (n_f_) treatment with an antibacterial agent, using the following equation:

4 $$\frac{{\mathrm{n}}_{\mathrm{i}}-{\mathrm{n}}_{\mathrm{f}}}{{\mathrm{n}}_{\mathrm{i}}}\times 100\mathrm{\%}.$$

For *E. coli* bacteria, as shown in Fig. 5a and b, after 30 min, bacteria started to be killed, and after 2 h, the percentage reduction of bacteria reached over 99% for samples containing Cu and Ti. However, for Ti samples alone, the percentage reduction of bacteria is less than 5% after 24 h. Based on the alterations curves in Fig. 5a, Ti_14_-Cu_86_ thin film has a steeper slope than the other samples, indicating a faster antibacterial process. Ti_29_-Cu_71_ and Ti_81_-Cu_19_ thin films have almost equal slopes and similar trends, while sample Ti_49_-Cu_51_ has the slowest bactericidal process compared to the other samples. Additionally, the visual evidence in Fig. 5b supports the antibacterial findings, showing photographs of agar plates with *E. coli* colonies after 30 min and 2 h of incubation. It can be seen that bacterial colonies decrease almost with the increase in Cu percentage. For *S. aureus* bacteria, which are shown in Fig. 5c and d, similar results were obtained. According to Fig. 5c, sample Ti_14_-Cu_86_ has the highest percentage of Cu, and has the highest bactericidal rate in 2 h, in contrast, samples Ti_29_-Cu_71_ and Ti_49_-Cu_51_ have almost the same trend, and sample Ti_81_-Cu_19_ lowest bactericidal rate. The percentage of bacterial reduction in the Ti sample alone is less than 5% in 2 h, while the samples containing Cu in contact with *S. aureus* bacteria are more than 99%. Figure 5a and c demonstrates that the presence of Ti layers alone resulted in a reduction of *S. aureus*, while no significant changes are observed in the case of *E. coli*. This discrepancy can be attributed to specific surface interactions^72^ and species-specific responses^73^ that play a crucial role in the antimicrobial activity of Ti layers against these two bacterial species. Moreover, *S. aureus* and *E. coli* possess distinct defense mechanisms and adaptive responses, influencing their susceptibility to the antimicrobial effects of the Ti layers^74^.

The mechanism through which Cu exerts its antimicrobial effects is multifaceted and involves several key factors^16,75^. One of the primary mechanisms consists of the release of Cu ions from the surface^76^. Cu exists in the body and biological environments in two oxidation states Cu^1+^ and Cu^2+^. Cu^1+^ ions are the most active form of Cu ions, and reducing the number of Cu^2+^ ions allows them to enter the bacterial membrane^77^. When the number of Cu^2+^ ions decreases, Ti-Cu thin films better fulfill their role as a killing agent against target bacteria. Because of Cu^1+^ ions play the main role in killing bacteria in our Ti-Cu thin films. The absorption of Cu^1+^ ions pierces the membrane, and the secretions resulting from this eventually kill the bacteria. The reduction of Cu^2+^ ions is done by performing the following reactions^78^. These reactions occur when Ti-Cu thin films are exposed to bacteria during incubation conditions.

5 $${\mathrm{Cu}}^{2+}+6{\mathrm{H}}_{2}\mathrm{O}\leftrightarrow {[\mathrm{Cu}{({\mathrm{H}}_{2}\mathrm{O})}_{6}]}^{2+},$$

6 $${[{\mathrm{Cu}\left({\mathrm{H}}_{2}\mathrm{O}\right)}_{6}]}^{2+}+ 2{\mathrm{H}}_{2}\mathrm{N}- {\mathrm{CH}}_{2}- {\mathrm{COO}}^{-} \to \left[{\mathrm{Cu}}^{2+}{\left({\mathrm{H}}_{2}\mathrm{N}-{\mathrm{CH}}_{2}-{\mathrm{COO}}^{-}\right)}_{2}{\left({\mathrm{H}}_{2}\mathrm{O}\right)}_{2}\right]+4{\mathrm{H}}_{2}\mathrm{O}.$$

The explanation states that the amino acids present in the environment during the incubation process react with Cu^2+^ ions according to the chemical equations mentioned above, leading to their consumption. These equations demonstrate that the amino acids in the environment facilitate the release of Cu^2+^ ions from the surface of Ti-Cu thin films, allowing them to interact with the surrounding environment. As a result, Cu^1+^ ions remain on the surface of the layers. Finally, these ions are in direct contact with the bacteria, are absorbed by its outer membrane, and react with this membrane according to the electronegative tendencies of the element. This causes a hole in the bacterial membrane, ultimately leading to its demise^79^.

Additionally, Cu can generate reactive oxygen species (ROS) through redox reactions. ROS, such as hydrogen peroxide and hydroxyl radicals, possess strong oxidizing properties that can damage bacterial cells. The production of ROS by Cu further enhances its antimicrobial activity^76^. Moreover, Cu has been found to interfere with biofilm formation, which plays a crucial role in bacterial resistance. Cu ions disrupt the extracellular matrix of biofilms, preventing their formation and rendering them more susceptible to antimicrobial agents^80^. In our study, we observed that incorporation Cu into Ti surfaces enhanced their antibacterial properties compared to Ti alone.

As we know, the Gram-positive bacteria membrane is thicker than the Gram-negative bacteria membrane^81^. Since the weight percentage of Cu and then the concentration of Cu^1+^ ions^82^ (as an antibacterial agent) are the same in Ti_81_-Cu_19_ thin films against these two kinds of bacteria, in Fig. 5a, it can be observed that these layers effectively fulfill their antibacterial role as a killer agent in the exposure of *E. coli* bacteria, similar to other layers. This behavior shows that the given amount of Cu and the concentration of Cu^1+^ ions is sufficient to eliminate all *E. coli* bacteria in the environment. However, as seen in Fig. 5c, Ti_81_-Cu_19_ thin films do not exhibit the same efficiency against *S. aureus* bacteria compared to other layers. This reduction efficiently can be attributed the thicker membrane of *S. aureus* bacteria when exposed to these layers. This can be explained by the fact that all the Cu^1+^ ions in the environment, when exposed to the thick membrane of *S. aureus* bacteria, are consumed and unable to eliminate them. Therefore, as seen in the CFU/ml analysis graph, we observe a bacteriostatic behavior. Figure 5d confirms these results with photographs of plates at 30 min and 2 h for *S. aureus* bacteria. After only 30 min of contact, a significant antibacterial effect was observed. Based on the obtained results, it is possible to see the prominent role of Cu metal compared to Ti metal in its antibacterial properties.

This was evident through the notable reduction in bacterial viability and observable physical deformation of bacterial cells on the Ti-Cu films. The images related to *E. coli* and *S. aureus* antibacterial agar plate counting test at 6 h and 24 h for each thin film can be found in Figs. 7S and 8S of Supplementary Information, respectively.

**Figure 6:**
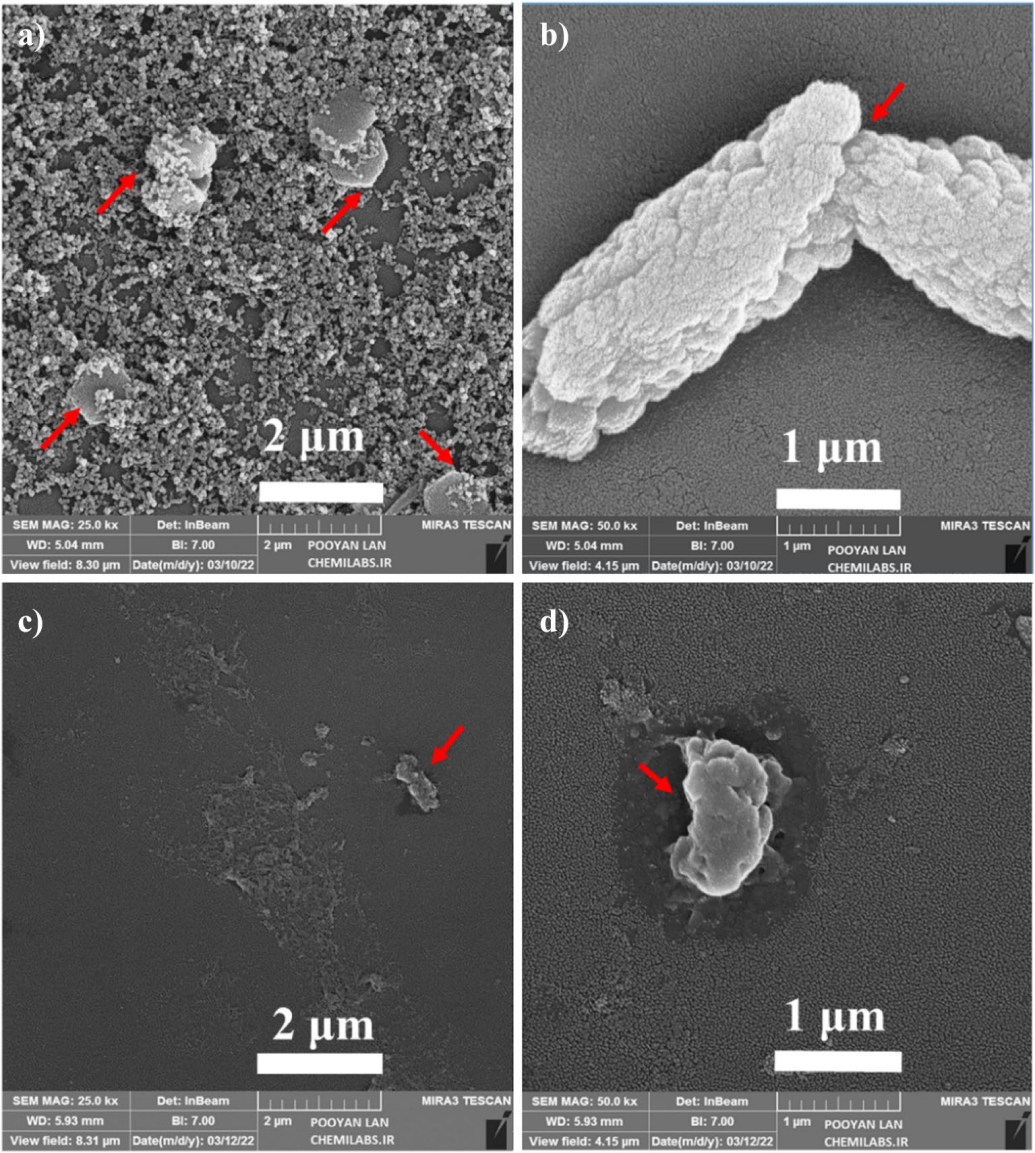
FESEM images of *E. coli* bacteria in the presence of Ti_49_-Cu_51_ thin film in scale (**a**) 2 μm (**b**) 1 μm and Ti_14_-Cu_86_ thin film in scale (**c**) 2 μm (**d**) 1 μm.

**Figure 7:**
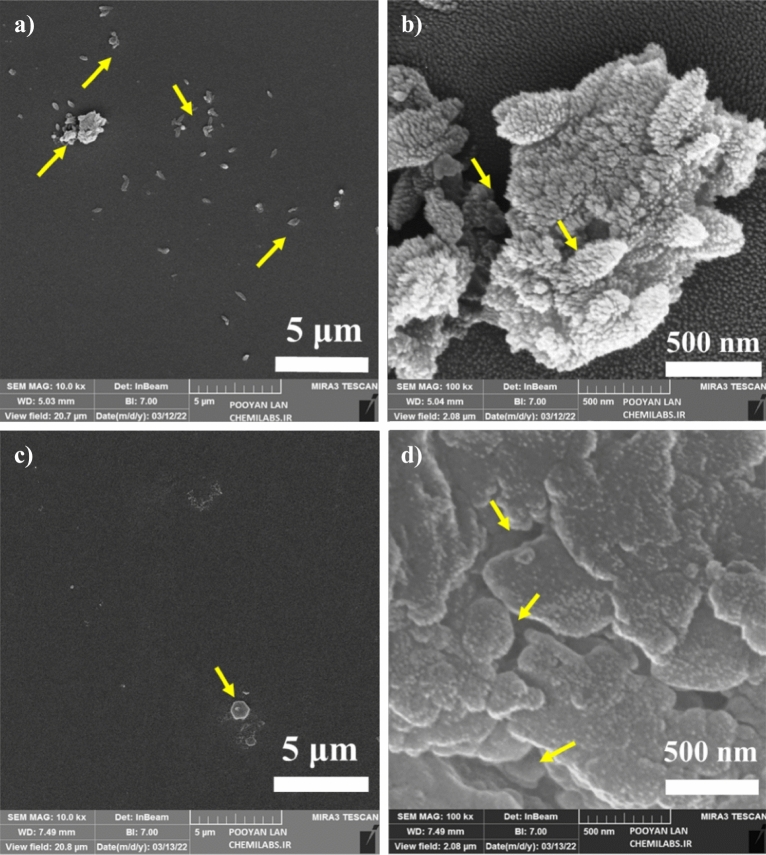
FESEM images of S. *aureus* bacteria in the presence of Ti_49_-Cu_51_ thin film in scale (**a**) 5 μm (**b**) 500 nm and Ti_14_-Cu_86_ thin film in scale (**c**) 5 μm (**d**) 500 nm.

In addition to CFU/ml analysis, FESEM images were used for the antibacterial assay of thin films, as depicted in Figs. 6 and 7. It is important to note that CFU/ml analysis not only measures bacterial quantity but also serves as a determinant of bacterial viability. The inclusion of CFU/ml analysis, along with FESEM images, in our antibacterial assay offers a holistic approach for assessing both quantitative viability measurements and visually confirming bacterial presence and structure. As shown in Fig. 6a–d, FESEM images demonstrate the morphological changes of *E. coli* bacteria on Ti_49_-Cu_51_ and Ti_14_-Cu_86_ thin films at different resolutions. The Ti_49_-Cu_51_ thin film in Fig. 6a, several bacteria with relatively damaged membranes are visible, indicated by red arrows. However, in the higher-resolution FESEM images in Fig. 6b, the perforated membrane of *E. coli* bacteria is more prominently highlighted by the red arrow. The Ti_14_-Cu_86_ thin film, which has the highest percentage of Cu among the samples, Fig. 6c shows that the bacteria are mostly killed, with the degraded membranes indicated by a red arrow. In the higher-resolution Fig. 6d, the degradation of the *E. coli* membrane after 30 min is depicted, as indicated by the red arrow. Based on the CFU/ml results and FESEM images, it can be concluded that the Ti_14_-Cu_86_ sample, with the highest percentage of Cu among the samples, exhibits the best antibacterial performance.

Figure 7a–d displays FESEM images of *S. aureus* bacteria on Ti_49_-Cu_51_ and Ti_14_-Cu_86_ samples after 30 min exposure, captured at different resolutions. In Fig. 7a, some *S. aureus* bacteria with a damaged membrane remain on the Ti_49_-Cu_51_ sample, indicated by the yellow arrow. Figure 7b, at the higher resolution, reveals bacteria with perforated and destroyed membranes. In Fig. 7c and d, for the Ti_14_-Cu_86_ sample, destroyed membrane of *S. aureus* bacteria at two resolutions, respectively, indicated by the yellow arrow. Only a few bacteria with damaged membrane can be seen in Fig. 7c and d, which shows the excellent antibacterial rate of Ti_14_-Cu_86_ thin film compared to other samples. FESEM images of the control, related to *E. coli* and *S. aureus* bacteria, are presented in Figs. 9S and 10S in the Supplementary file, respectively. These images effectively show the expected appearance and structure of the cells under favorable conditions, confirming that the sample preparation process for FE-SEM did not cause any detrimental effects on the bacterial cells. The raw data of the FESEM image is given in Figs. 11S and 12S in the Supplementary file.

### Topography of deposit thin films

**Figure 8:**
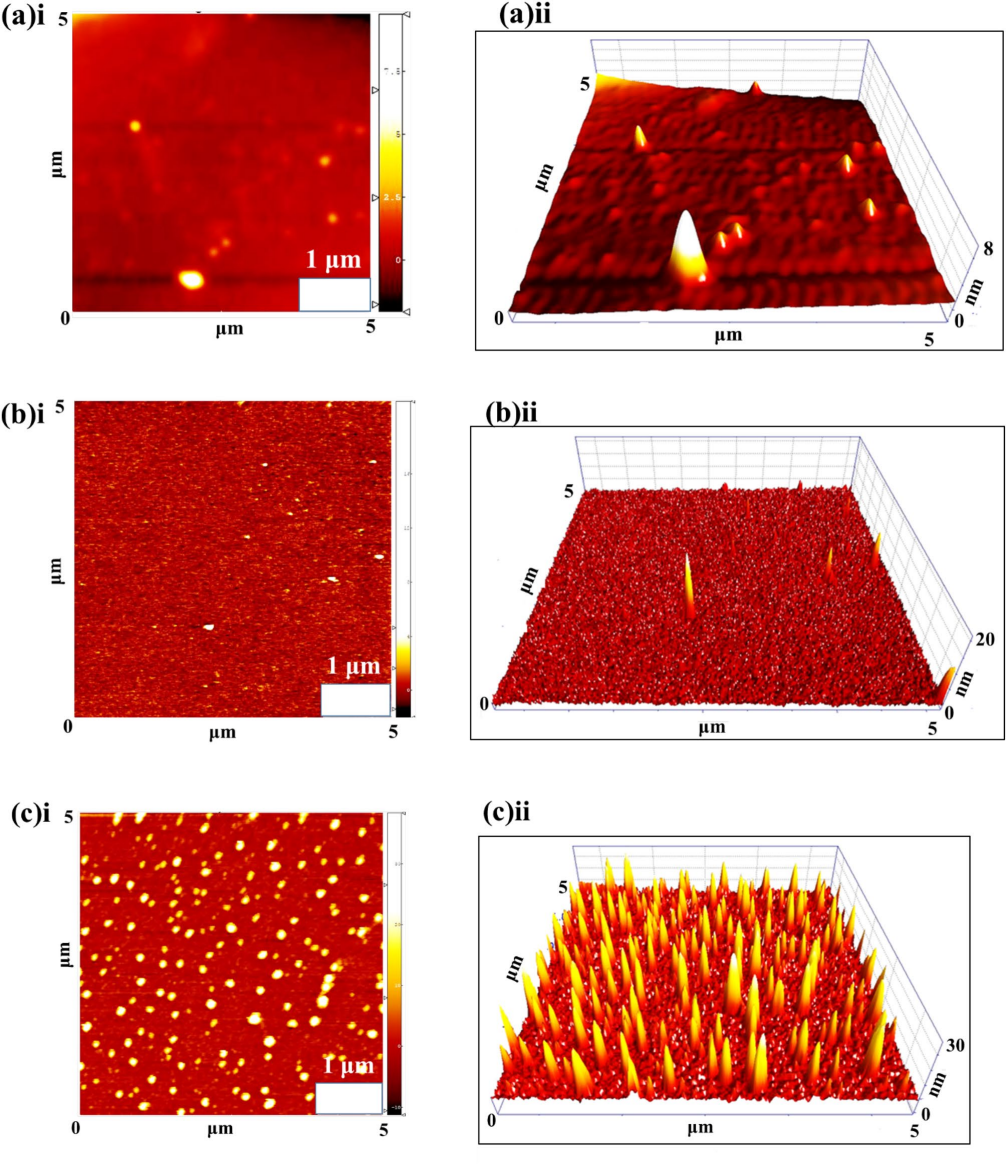

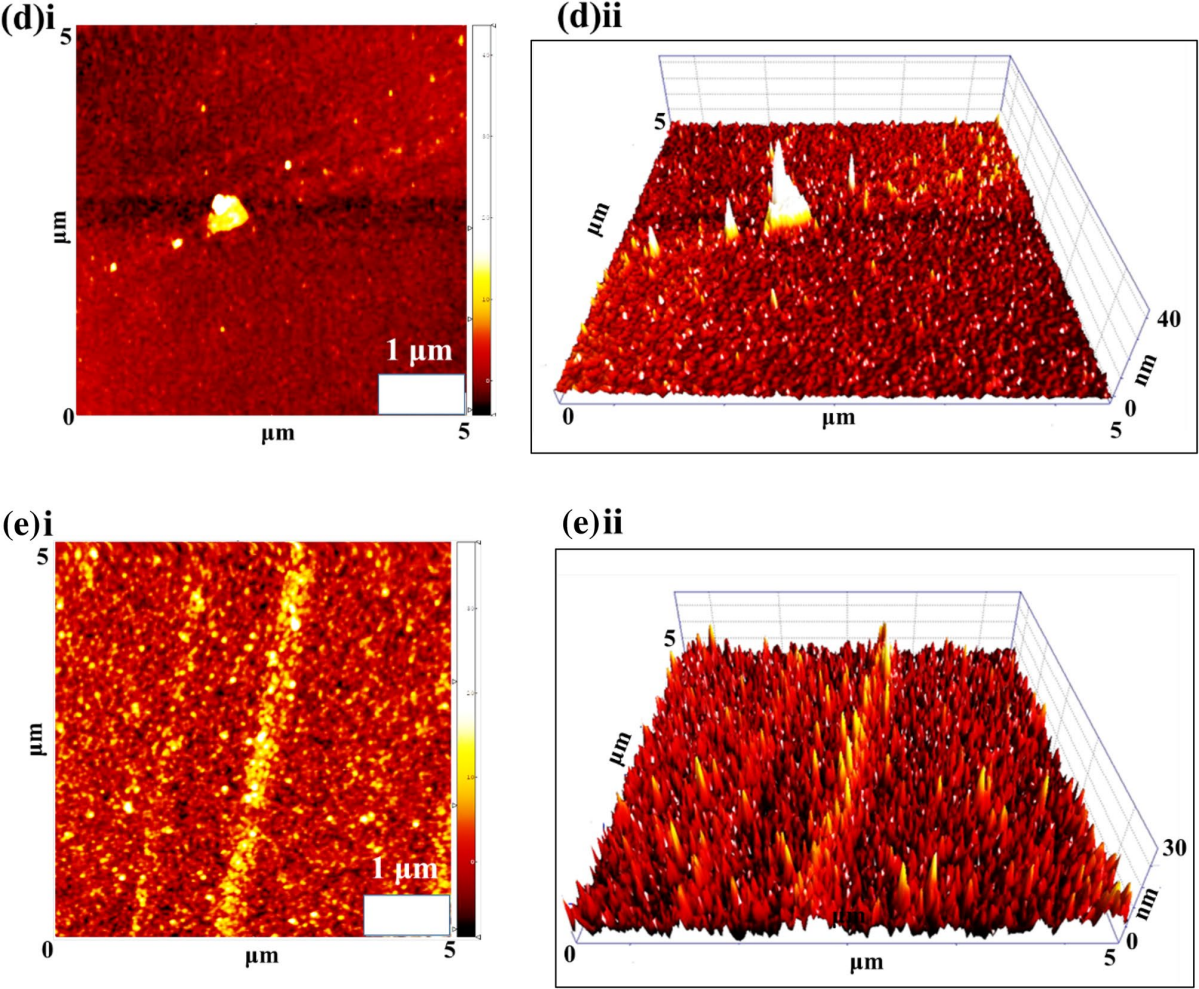
AFM topographic 2D and 3D maps of (**a**) Ti (**i**,**ii**), (**b**) Ti_81_-Cu_19_ (**i**,**ii**), (**c**) Ti_49_-Cu_51_(**i**,**ii**), (**d**) Ti_29_-Cu_71_(**i**,**ii**), and (**e**) Ti_14_-Cu_86_ (**i**,**ii**) thin films.

The surface topography and coating quality of the prepared thin films were investigated using AFM imaging. Figure 8a–e demonstrated AFM topographic 2D and 3D maps of the prepared thin films. The representative 3D maps of the thin film surface confirm the homogeneity and lack of cracks. It can be observed that the surface roughness increases relative to the amount of Cu. The roughness RMS parameters were calculated within a small surface area (5 × 5 µm^2^). The Ti, Ti_81_-Cu_19_, Ti_49_-Cu_51_, Ti_29_-Cu_71_, and Ti_14_-Cu_86_ coatings have RMS values of 11.85, 23.42, 49.65, 47.99, and 46.81 nm, respectively. In this study, Ti_14_-Cu_86_, Ti_29_-Cu_71,_ and Ti_49_-Cu_51_ samples, depicted in Fig. 8c, d, and e, respectively, exhibit roughness between 46 and 49 nm and demonstrate better antibacterial properties compared to Ti and Ti_81_-Cu_19_ samples, which have a roughness of 11–23 nm shown in Fig. 8a and b, respectively. To further elucidate the demonstrated topography across the surface and provide visual representations of the surface features, line profiles are included in the Supplementary file (Figs. 13S-17S).

**Figure 9:**
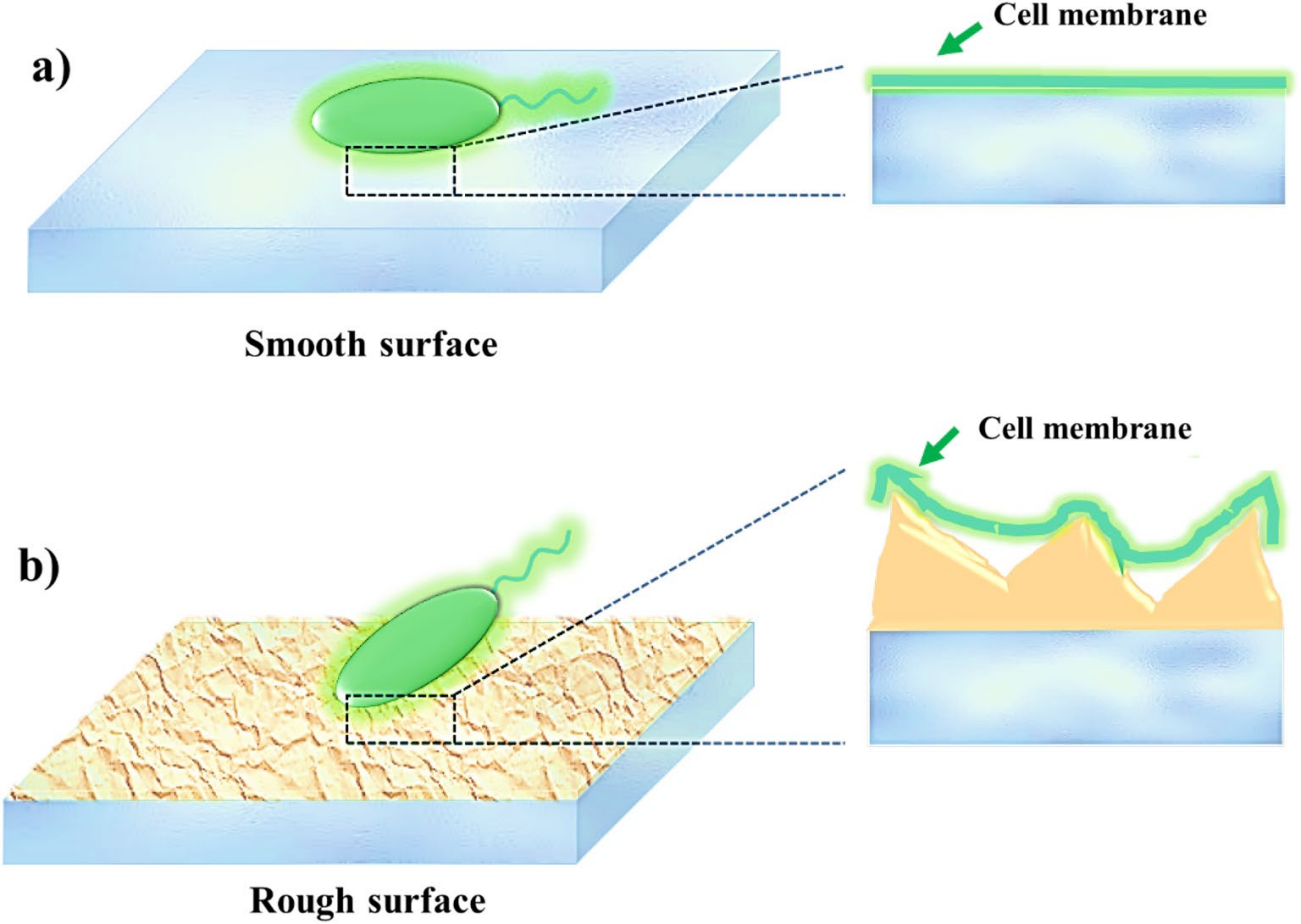
Schematic views of the cell membrane contact with (**a** (the smooth surface and (**b**) surface with nanoscale roughness.

Surfaces that exhibit nanoscale roughness, particularly when combined with a sufficient amount of antibacterial agents such as Cu, offer potential benefits for antibacterial applications. As shown in schematic Fig. 9a, when the surface is smooth and lacks roughness, bacteria naturally adhere to it. However, when the surface features a nanoscale roughness structure, as depicted in Fig. 9b, bacteria tend to have increased contact with the surface. Increasing contact surface can lead to elongation and rupture of the cell membrane, ultimately destroying the bacteria. Consequently, surfaces with higher nanoscale roughness can inhibit cell proliferation and biofilm formation due to the alterations in the shape of the bacterial membrane^83^.

### Contact angel

**Figure 10:**
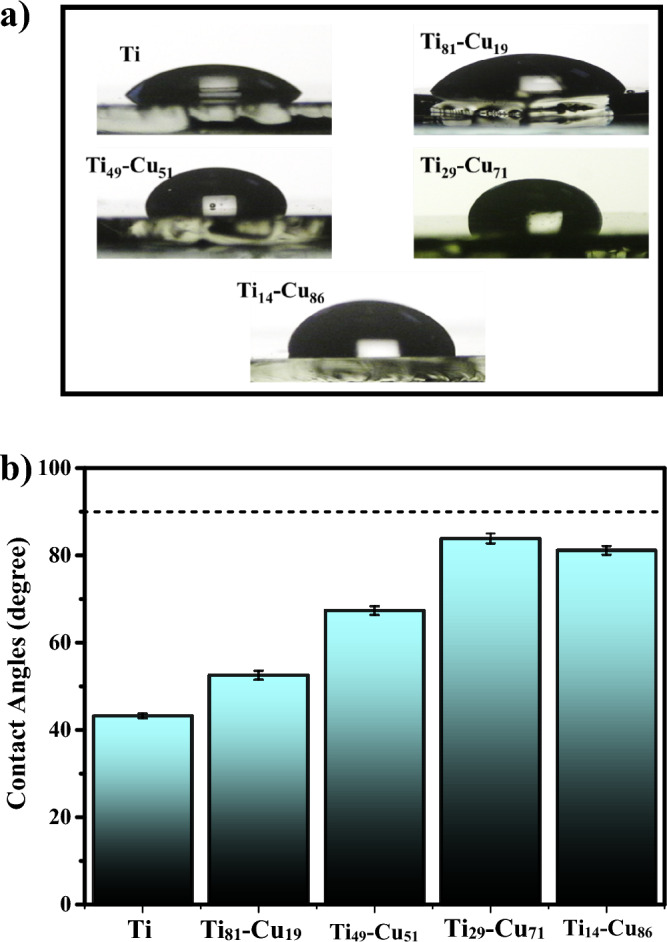
(**a**) Sessile drop contact angle measurement of deionized water on the prepared thin films on a glass surface (**b**) Results of the contact angle measurements.

As shown in Fig. 10a and b, the water contact angles of prepared thin films on the glass substrate were determined using the sessile drop method. As observed, the Ti thin layer exhibited a lower contact angle of about 40 degrees. In contrast, the contact angle of the sample Ti_14_-Cu_86_ with a higher percentage of Cu increased to 80 degrees. The 90-degree cut-off point for hydrophobicity is depicted by the horizontal line in Fig. 10b. Any value above this line indicates a hydrophobic surface. In contrast, values below it denote a hydrophilic surface. It was observed that the Ti thin film exhibited a contact angle of about 40 degrees, indicating a hydrophilic surface. In contrast, the contact angle increased to around 80 degrees with a higher percentage of Cu in Ti-Cu thin films, indicating a tendency to a hydrophobic behavior. Additionally, it was noted that the antibacterial properties of the thin film improved with an increase in Cu percentage. This finding indicates that increasing the Cu content, acting as an antibacterial agent, leads to an increase in the contact angle, effectively reducing bacterial adherence to the surface.

### Biocompatibility activities of Ti-Cu thin films

**Figure 11:**
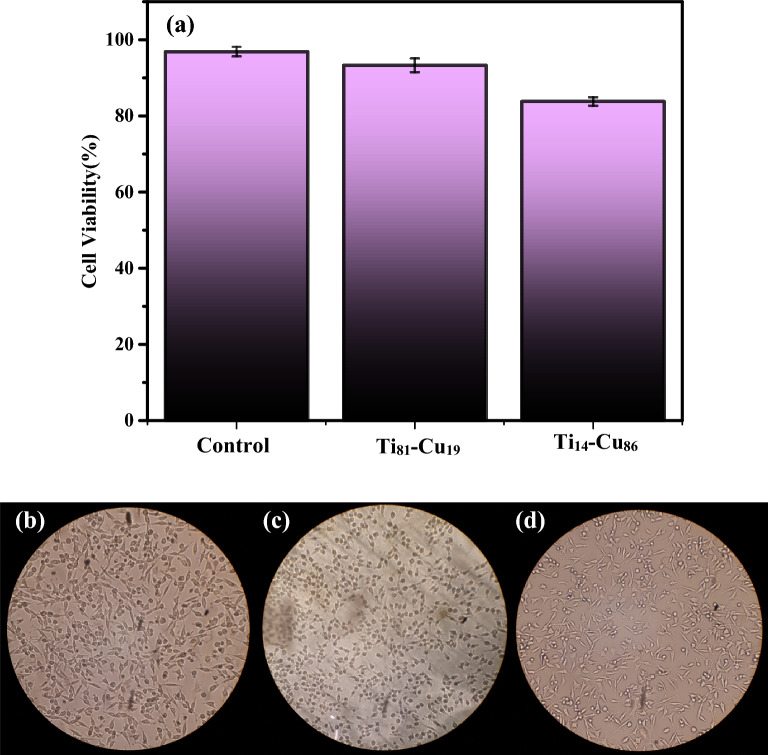
(**a**) the outcomes of the MTT test and images of cells after exposure to the (**b**) control, (**c**) Ti_81_-Cu_19_, (**d**) and Ti_14_-Cu_86_ samples following a 24 ± 2 h period (n = 3). The average value of each parameter was evaluated using Duncan’s method (P < 0.05).

To assess the potential cytotoxicity of Cu-Ti thin films, an MTT test was conducted on L929 cells cultured with the thin films for 24 ± 2 h. The results, as illustrated in Fig. 11a–d, according to cell viability exceeding 70% across all experimental groups, no evidence of cytotoxicity demonstrated. Specifically, L929 cells exhibited an average survival rate of 93.28% on Ti_81_-Cu_19_ and 83.80% on Ti_14_-Cu_86_ after 24 ± 2 h of culture. The difference in survival and proliferation between the Ti_81_-Cu_19_ and Ti_14_-Cu_86_ samples, representing the lowest and highest percentages of Cu, respectively, was only 10%. It is believed that these samples while killing the bacteria, at the same time they release no cytotoxic particulate debris from the thin films, indicating that the magnetron sputtered specimens used in this study are biocompatible.

Furthermore, it is important to note that both Ti and Cu are commonly employed metals in various medical applications. Extensive studies have examined the potential toxicity of Ti in human cells to confirm its safety. These investigations consistently demonstrate that Ti is generally non-toxic and suitable for human applications^84^. On the other hand, Cu is an essential element crucial for various biological processes, including enzymatic activity, redox reactions, and cellular signaling within the body^85^. Considering the dimensions of the samples, which consisted of nanoscale thin layers with a weight percentage of Cu, a 1 × 1 cm^2^ sample coated with 200 nm Ti_14_-Cu_86_ (having the highest weight percentage of Cu at 85.97% among the samples) contains 0.017 mg of Cu according to the weight percentage relationship. The literature suggests that the natural presence of Cu in an adult human weighing approximately 70 kg ranges from 75 to 100 mg^78^. These findings provide substantial evidence supporting the safe use of Ti-Cu-based materials in the context of biomaterials.

## Discussion

In this study, we describe the fabrication of a bactericidal surface through the combination of Ti and Cu. Our approach involves a powerful Co-sputtering method, allowing us to tailor the antibacterial properties of the surface by adjusting the relative proportions of Ti and Cu. The findings of this study contribute to the ongoing efforts to develop effective antibacterial strategies and combat antibiotic resistance. Traditional antibiotics are losing effectiveness due to the adaptation and proliferation of resistant strains, making it imperative to explore alternative approaches to combat bacterial infections^86^.

Testing materials for their antimicrobial and antibiofilm properties is crucial, particularly in the context of combating bacterial infections and antibiotic resistance^87^. When assessing antimicrobial activity, researchers typically conduct tests to evaluate the efficacy of a substance or material inhibiting growth bacteria. These tests involve exposing bacteria to the material and measuring how much bacterial growth is inhibited. On the other hand, evaluating antibiofilm activity involves evaluating a substance’s ability to prevent or disrupt biofilm formation. This is essential because biofilms exhibit high resistance to antibiotics^88^. Various techniques are used to test antibiofilm properties, including quantifying biofilm biomass, measuring the viability of bacteria within the biofilm, and assessing the structural integrity of the biofilm matrix^89,90^. These tests play a crucial role in developing new materials, coatings, or compounds to combat bacterial infections, particularly those associated with biofilms. Biofilms, which involve bacteria enclosed in a protective extracellular matrix, are highly resistant to antibiotics^91,92^. This resistance can be attributed to two factors. First, the biofilm matrix acts as a physical barrier, restricting the penetration of antibiotics and limiting their effectiveness. Additionally, the dense structure of biofilms hinders the diffusion of drugs, further reducing their ability to reach all the bacterial cells^93,94^. Second, biofilms contain a subpopulation of bacteria known as persister cells, which exhibit a dormant or slow-growing state that renders them highly tolerant to antibiotics. Once the antibiotic treatment ceases, persister cells can revitalize and repopulate the biofilm, leading to recurrent infections^95^.

Surface modification has emerged as a promising strategy to inhibit biofilm formation and bacterial adherence in implants. By altering the properties of surfaces, such as their composition or topography, it is possible to create antibacterial surfaces that prevent bacterial colonization and growth. Ti_14_-Cu_86_ thin film has demonstrated exceptional antibacterial performance, surpassing many other Cu and Ti-based coatings, as shown in Table 3. This outstanding antibacterial efficacy can be attributed to the unique structure and the relative Cu-Ti ratio of the films. These findings highlight the potential of Ti_14_-Cu_86_ thin film for various biomaterial applications, including dental and bone implants, due to their antibacterial properties. When comparing our results with the existing literature, significant improvements in antibacterial performance have been achieved. Notably, our Ti-Cu thin films reached 99.9% reduction bacteria, surpassing values reported for similar composites such as Cu and Ti additives^96^. Additionally, the thin films exhibited rapid antibacterial activity, outperforming previous Cu and Ti-based surfaces^97^. These results emphasize the effectiveness of the sputtering method in fabricating high performance layers for biodevices.

**Table 3:** Comparing this work with previous works in terms of conducted method and efficiency.

Coating	Conducted method	Bacteria	Ability	Ref
TiO_2_–Cu nanocomposite films	Chemical vapor deposition	*S. aureus*	70% antibacterial activity	^96^
Films of titania and copper oxide	Chemical vapor deposition	*E. coli*	Self-cleaning and biocidal films	^98^
TiO_2_ thin films co-doped with Cu–Ce	Dip-coating technique	*E. coli and S. aureus*	Antibacterial ability	^99^
TiO_2_/CuO coating	Magnetron sputtering	*S. aureus*	99% antibacterial rate	^100^
Cu_2_O-TiO_2_/Ti_2_O_3_/TiO coating	Plasma-enhanced chemical vapor deposition	*S. aureus*	Antibacterial and endothelialization ability	^101^
Inter-metallic Ti–Cu films	Magnetron sputtering	*S. epidermidis and S. aureus*	Cytotoxic effect on osteoblasts	^102^
Cu_x_O/TiO_2_ coating	plasma electrolytic oxidation	*S. aureus*	Antibacterial activity	^97^
Ti_14_-Cu_86_ thin film	Co-sputtering	*E. coli and S. aureus*	99.9% reduction of bacteria in 2 h	This work

## Conclusions

This work presents a practical strategy, for preparing high-performance antibacterial Ti-Cu surfaces. The co-sputtering technique was employed to deposit antibacterial thin films within a thickness of 160-200nm on glass substrates. XRD spectroscopy showed an amorphous structure, and EDX confirmed the percentage of elements in each thin film. According to the CFU/ml analysis, a 99.9% decrease in bacteria was observed after 2 h of exposure to Gram-negative and positive bacteria strains using Ti-Cu thin films. Additionally, FESEM images confirmed the destruction of the cell membrane in both bacterial strains after only 30 min of exposure. The topography of AFM maps showed that Cu-containing thin films have more nanoscale roughness than bare Ti thin films. This increased roughness can effectively contribute to the destruction of bacterial cell membranes and inhibit bacterial adherence. Additionally, as the percentage of Cu increases, the contact angle increases, leading to improved antibacterial properties. This is because a higher contact angle on the surface makes it more difficult for bacteria to adhere, inhibiting their growth. Therefore, this study demonstrates that increasing the percentage of Cu can improve antibacterial activity by modifying surface properties such as roughness and wettability. The results of our study demonstrate the biocompatibility and lack of cytotoxicity of Cu-Ti thin films, further supporting their potential as safe and effective materials for biomedical applications. Given the significant challenges posed by infectious contamination, our study contributes to the expanding research on using the magnetron sputtering method to enhance antimicrobial activity on surfaces, presenting promising findings.

### Supplementary Information

Supplementary Information.

## Data Availability

All data generated or analyzed during this study are included in this submitted article (and its Supplementary Information files).
