# Supplementary material for: Improving antibacterial ability of Ti-Cu thin films with co-sputtering method
Source: Sci Rep. 2023 Oct 3;13:16593. doi: 10.1038/s41598-023-43875-4 (PMC10547835; doi:10.1038/s41598-023-43875-4)
Supplement: Supplementary file 1 — Supplementary Information. [file 41598_2023_43875_MOESM1_ESM.docx]

*Supplementary Information for:*

**Improving Antibacterial Ability of Ti-Cu Thin Films with Co-Sputtering Method**

Samaneh Mahmoudi-Qashqay^1^, Mohammad-Reza Zamani-Meymian^1^*, Seyed Javad Sadati^1^

*^1^Department of Physics, Iran University of Science and Technology, P.O. Box 16846-13114, Tehran, Iran*

**Corresponding author E-mail: Mohammad-Reza Zamani-Meymian,r_zamani@iust.ac.ir, mrzamanian@yahoo.com; Fax: +98 21- 7724 0497; Tel: +98-21-7322 5893*


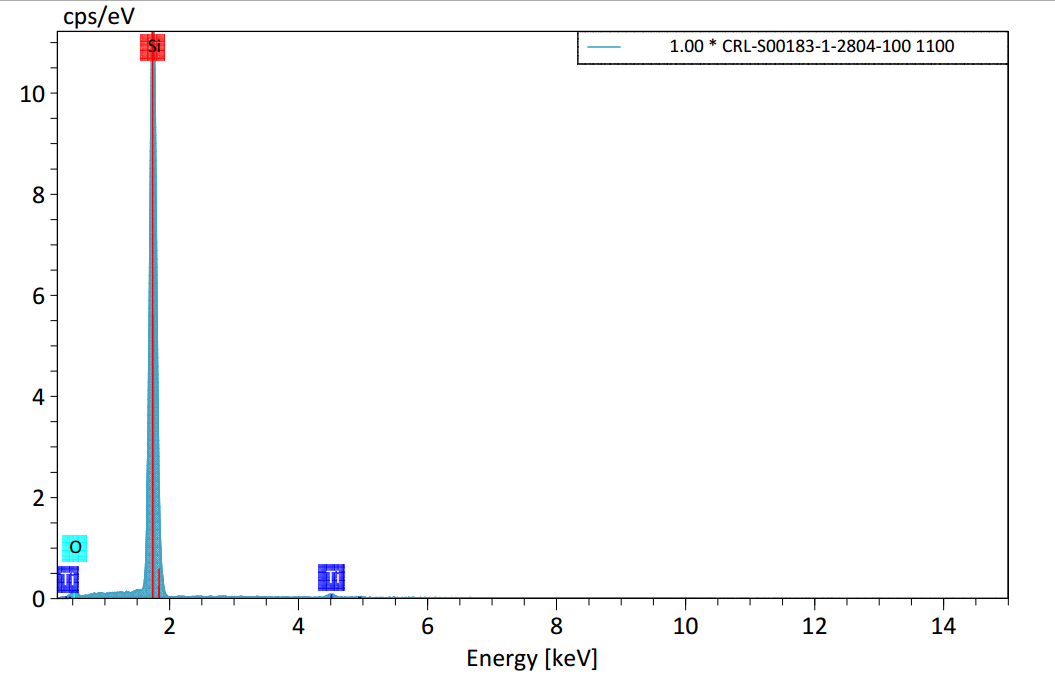


**Figure 1S.** EDX Analysis of the Ti thin film


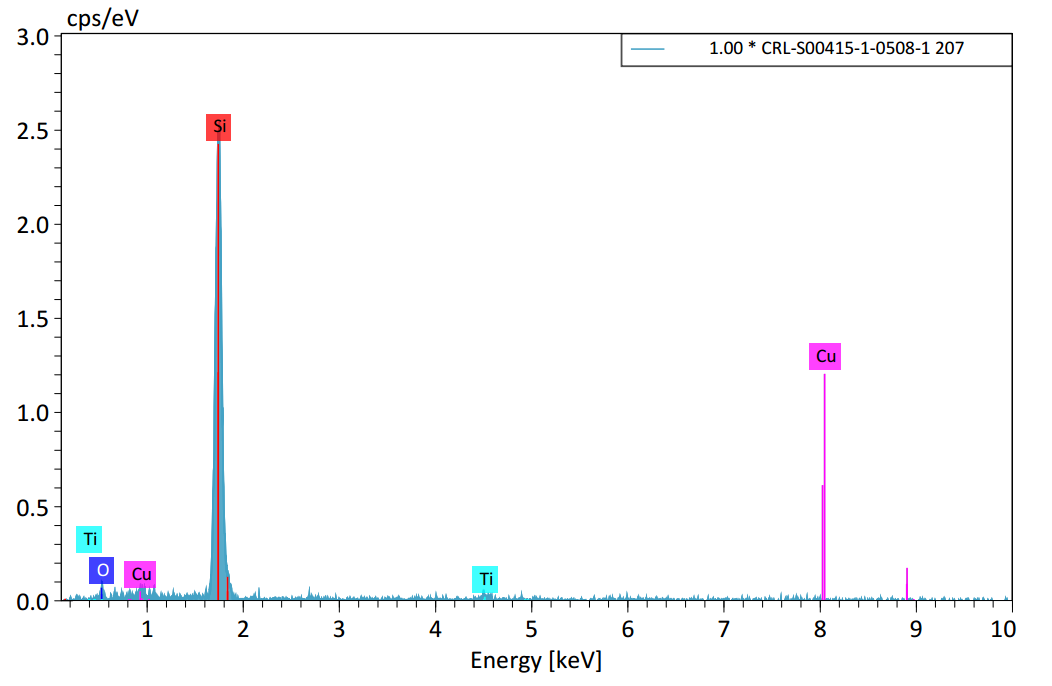


**Figure 2S.** EDX Analysis of the Ti_81_-Cu_19_ thin film


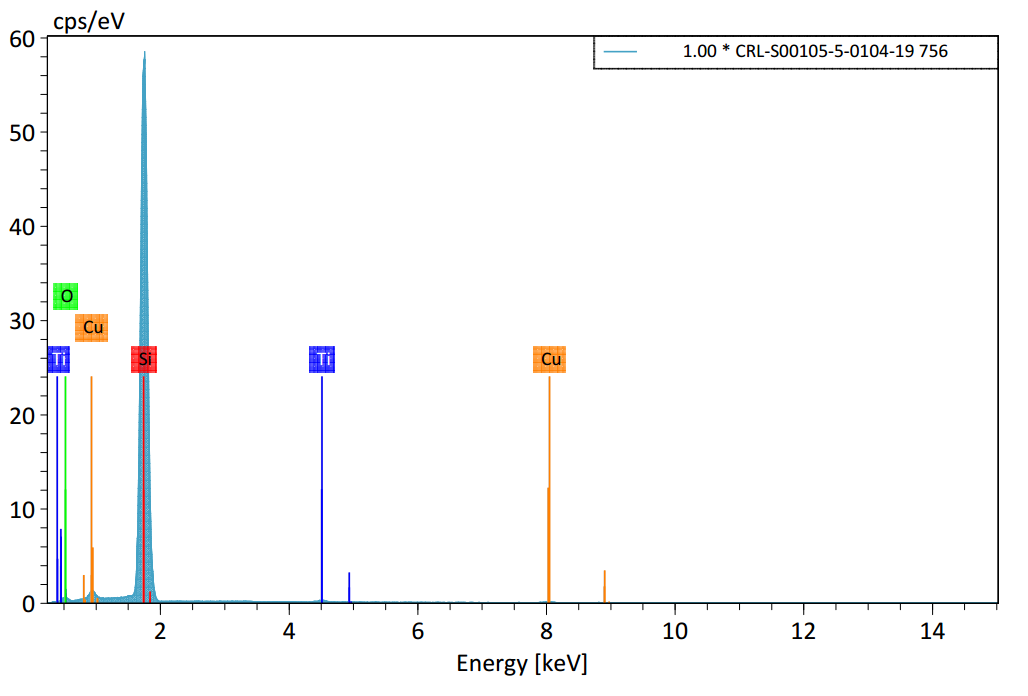


**Figure 3S.** EDX Analysis of the Ti_29_-Cu_71_ thin film


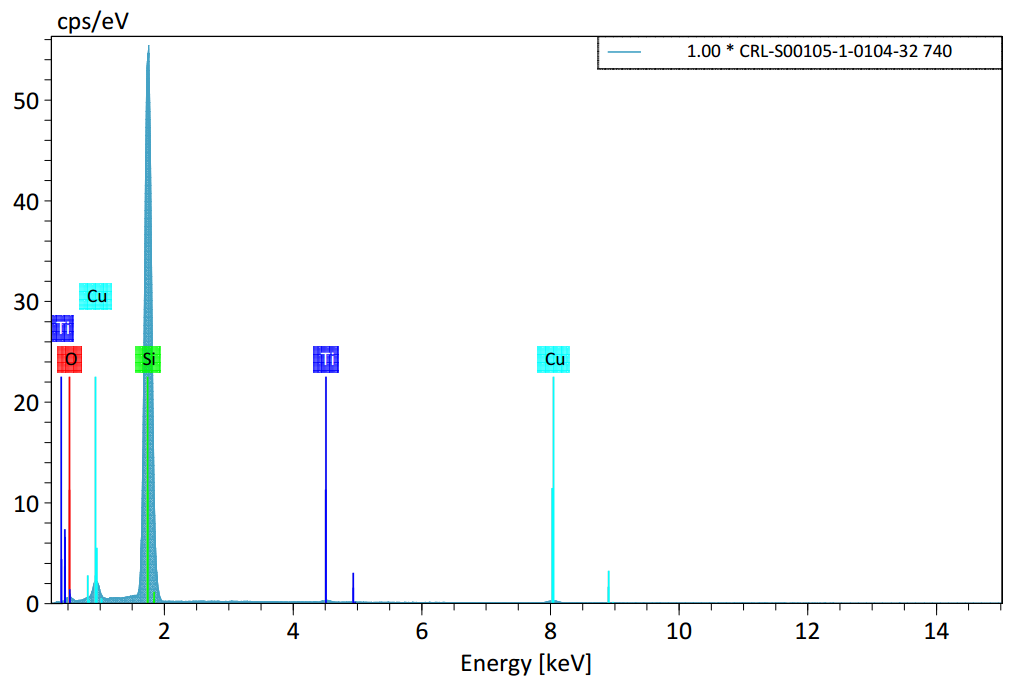


**Figure 4S.** EDX Analysis of the Ti_14_-Cu_86_ thin film

Table 1S. Mean ± Standard Deviation of mass and atomic percentages of elements in each thin film

|  | | Element | | |  | |  |  |
| --- | --- | --- | --- | --- | --- | --- | --- | --- |
| Ti | | Cu | | | Ti Cu | |  | |
| samples | | Mean $\pm$ Standard Deviation  %Mass | | | Mean $\pm$Standard Deviation  %Atom | |  |  |
| Ti | 100$\pm$0.000 | | 0.00$\pm$0.000 | 100$\pm$0.000 | | 0.00$\pm$0.000 |  |  |
| Ti_81_-Cu_19_ | 80. 87$\pm$0.120 | | 19.13$\pm$0.120 | 84.87$\pm$0.106 | | 15.12±0.106 |  |  |
| Ti_49_-Cu_51_ | 48.42$\pm$0. 601 | | 51.57$\pm$0. 601 | 44.36$\pm$1.170 | | 55.63$\pm$1.169 |  |  |
| Ti_29_-Cu_71_ | 27.08$\pm$1.378 | | 72.91$\pm$1.378 | 70.85$\pm$0.799 | | 29.15$\pm$0.678 |  |  |
| Ti_14_-Cu_86_ | 13.05$\pm$0.081 | | 86.95$\pm$0.081 | 16.96$\pm$0.844 | | 83.03±0.844 |  |  |


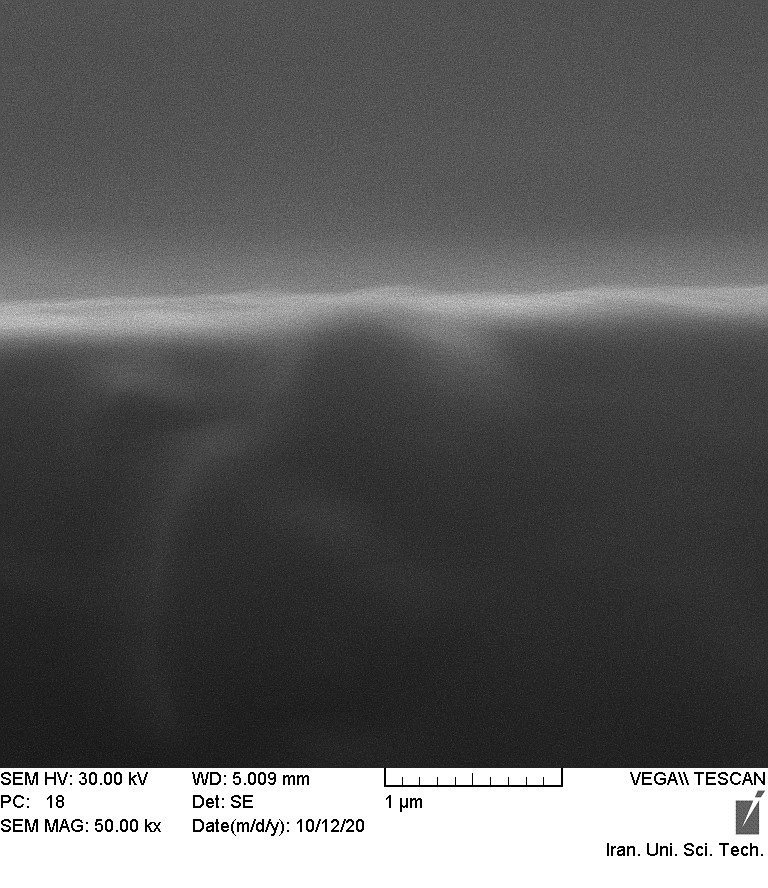


**Figure 5S.** The raw data of SEM image of the cross section view of the sample Ti_49_-Cu_51_.

**Ti**


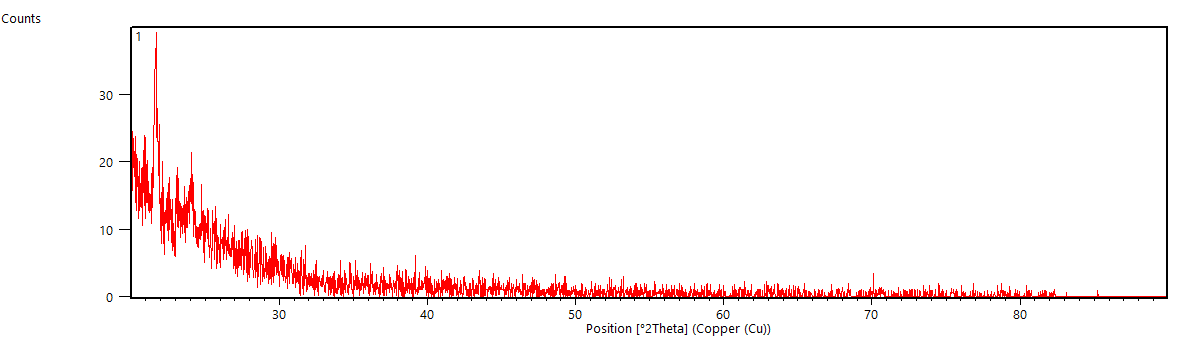


**Ti_81_-Cu_19_**


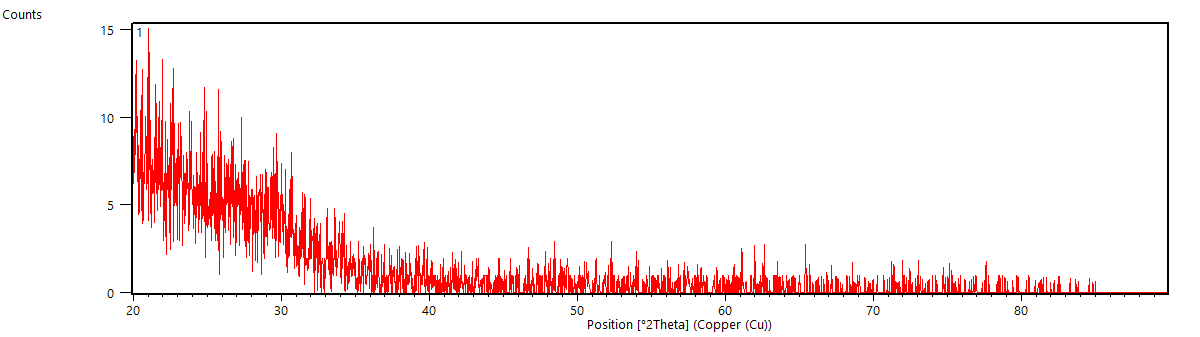


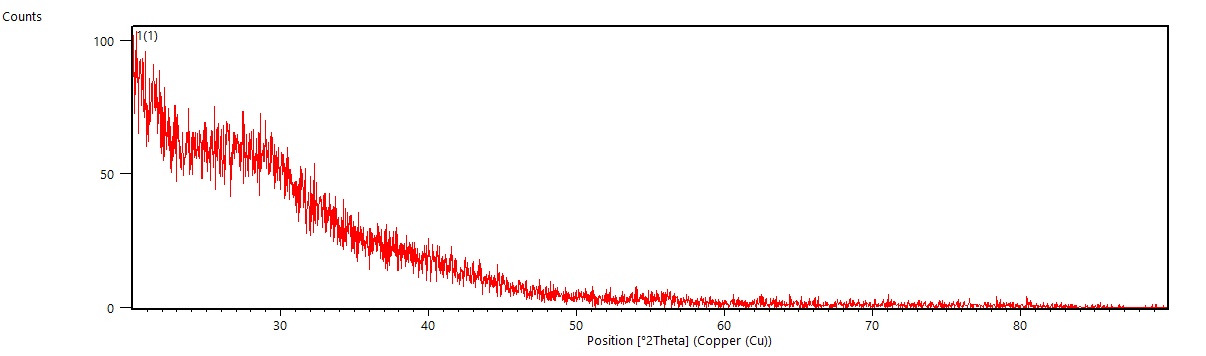


**Ti_29_-Cu_71_**

**Ti_49_-Cu_51_**


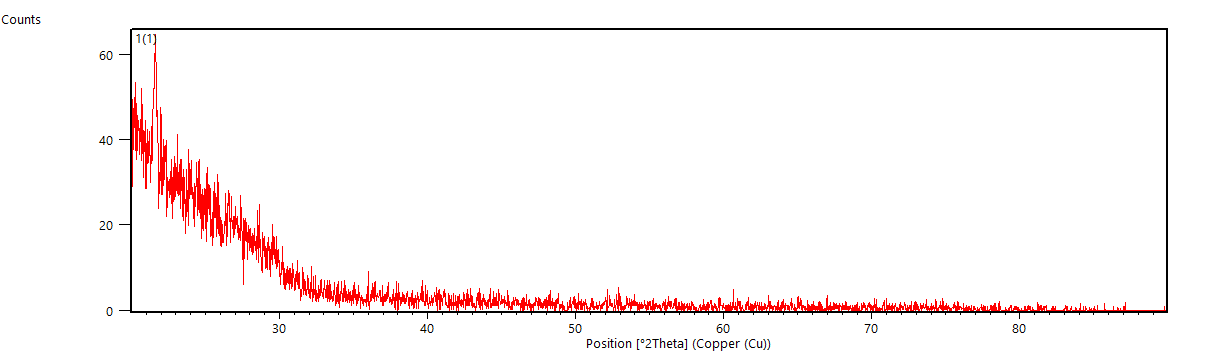


**Ti_14_-Cu_86_**


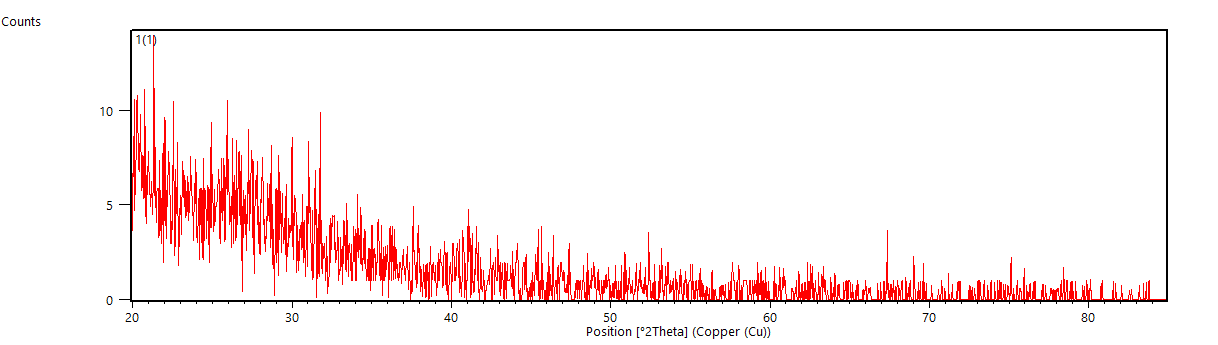


**Figure 6S.** The raw data of GIXRD pattern of the Ti-Cu thin films on the glass substrate.


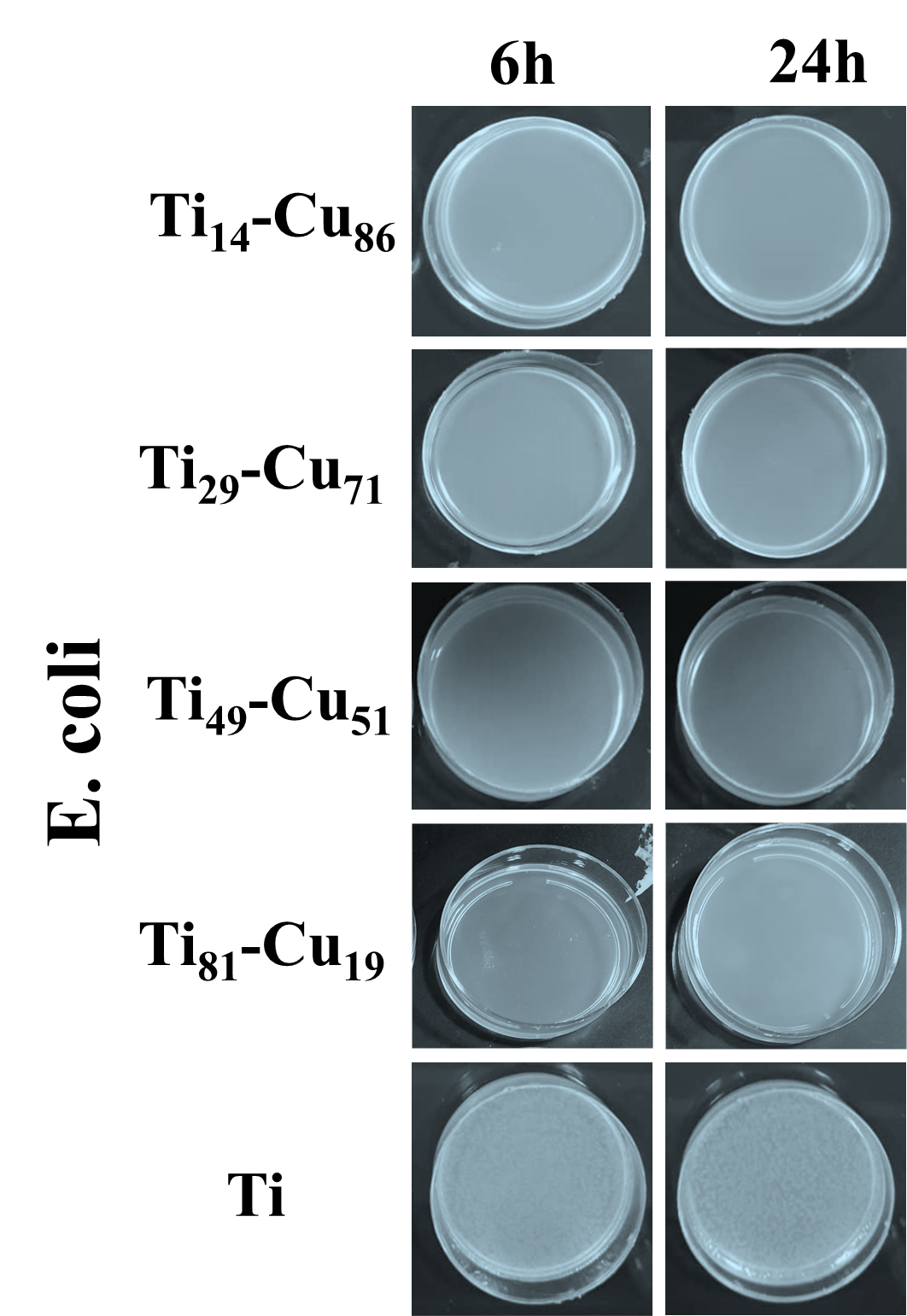


**Figure 7S.** Photo of plate count agar antibacterial assay of E. coli for each of thin films at 6h and 24h.


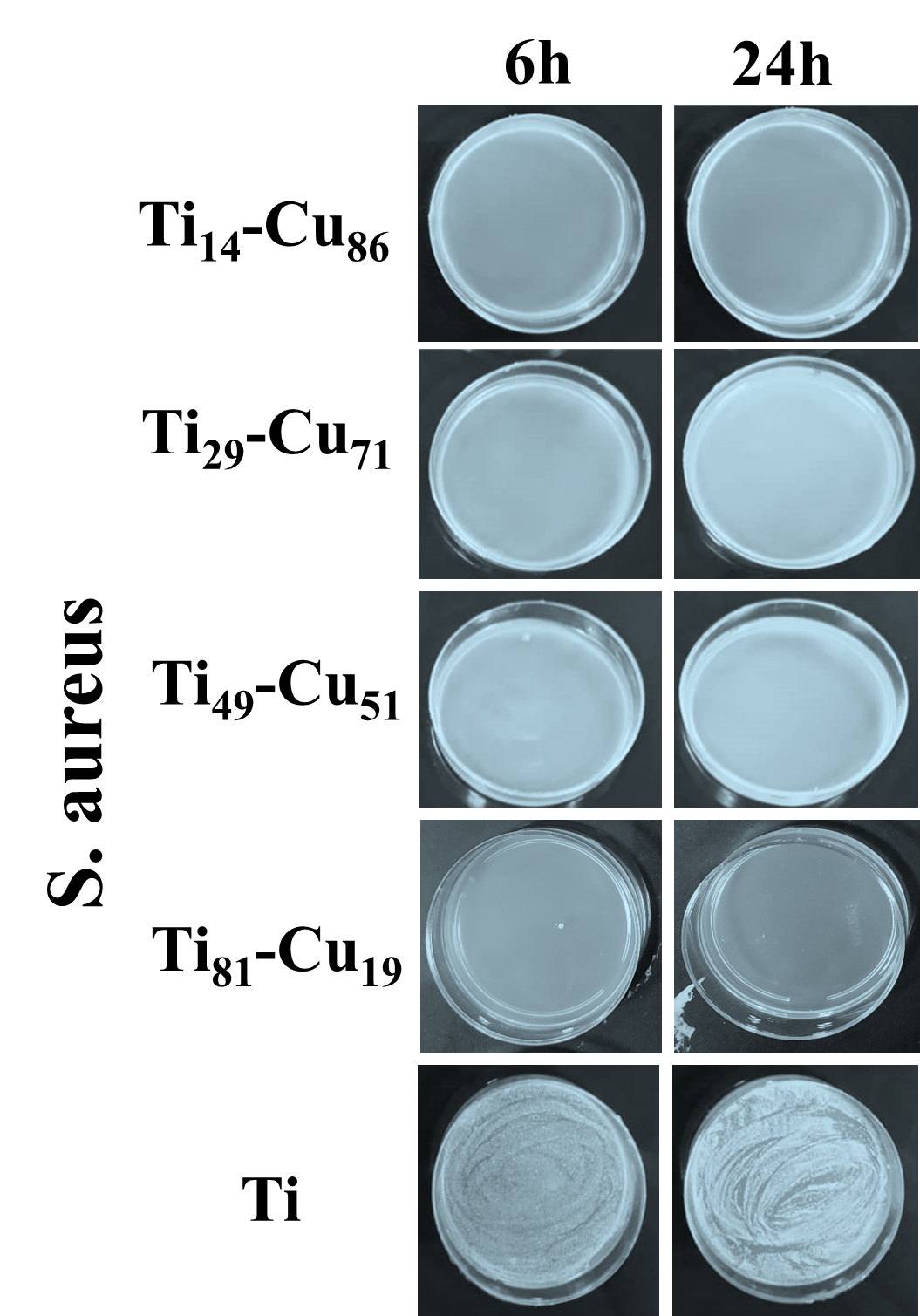


**Figure 8S.** Photo of plate count agar antibacterial assay of S. aureus for each of thin films at 6h and 24h.

Table 2S. Mean total viable count (TVC) bacteria count (CFUml^−1^) and (log10 CFUml^-1^) of Strain S. aureus in 30min, 2h, 6h, 24h

| samples |  |  |  | | Time | | | | | | | |  | |  |
| --- | --- | --- | --- | --- | --- | --- | --- | --- | --- | --- | --- | --- | --- | --- | --- |
|  | **30min** | **30min** | **2h** | **2h** | | | **6h** | | **6h** | | **24** | | **24h** | |  |
|  | Mean TVC  (CFUml^−1^) | Mean TVC (log10 CFUml*^-^*^1^) | Mean TVC  (CFUml^−1^) | Mean TVC (log10 CFUml*^-^*^1^) | | | Mean TVC  (CFUml^−1^) | | Mean TVC (log10 CFUml*^-^*^1^) | | Mean TVC  (CFUml^−1^) | | Mean TVC (log10 CFUml*^-^*^1^) | |  |
| Ti | 9.5 × 10^5^ | 0.022$\pm$0.061 | 9.5 × 10^5^ | | | 0.022$\pm$0.082 | | 9.5 × 10^5^ | | 0.022$\pm$0.032 | | 1 × 10^5^ | | 0.046$\pm$0.025 | |
| Ti_81_-Cu_19_ | 3.5 × 10^5^ | 0.456$\pm$0.056 | 2 × 10^3^ | 2.699$\pm$0.021 | | | 1 | | 5$\pm$0.011 | | 1 | | 5$\pm$0.028 | |  |
| Ti_49_-Cu_51_ | 1 × 10^5^ | 0.046$\pm$0.095 | 2 × 10^1^ | 4.699$\pm$0.036 | | | 1 | | 6$\pm$0.014 | | 1 | | 6$\pm$0.058 | |  |
| Ti_29_-Cu_71_ | 8.7 × 10^4^ | 0.060$\pm$0.025 | 9 | 5.046$\pm$0.087 | | | 1 | | 6$\pm$0.013 | | 1 | | 6$\pm$0.045 | |  |
| Ti_14_-Cu_86_ | 5 × 10^4^ | 1.301$\pm$0.012 | 2 | 5.699$\pm$0.0641 | | | 1 | | 6$\pm$0.017 | | 1 | | 6$\pm$0.026 | |  |

Table 3S. Mean total viable count (TVC) bacteria count (CFUml^−1^) and (log10 CFUml^-1^) of Strain E. coli in 30min, 2h, 6h, 24h

| samples |  |  |  | | Time | | | | | | | |  | |  |
| --- | --- | --- | --- | --- | --- | --- | --- | --- | --- | --- | --- | --- | --- | --- | --- |
|  | **30min** | **30min** | **2h** | **2h** | | | **6h** | | **6h** | | **24h** | | **24h** | |  |
|  | Mean TVC  (CFUmL^−1^) | Mean TVC (log10 CFUmL*^-^*^1^) | Mean TVC  (CFUmL^−1^) | Mean TVC (log10 CFUmL*^-^*^1^) | | | Mean TVC  (CFUmL^−1^) | | Mean TVC (log10 CFUmL*^-^*^1^) | | Mean TVC  (CFUmL^−1^) | | Mean TVC (log10 CFUmL*^-^*^1^) | |  |
| Ti | 9.5 × 10^5^ | 0.022$\pm$0.015 | 9.5 × 10^5^ | | | 0.022$\pm$0.019 | | 9.5 × 10^5^ | | 0.022$\pm$0.013 | | 9.5 × 10^5^ | | 0.022$\pm$0.011 | |
| Ti_81_-Cu_19_ | 7 × 10^4^ | 1.155$\pm$0.025 | 10 | 5$\pm$0.016 | | | 10 | | 5$\pm$0.018 | | 10 | | 5$\pm$0.029 | |  |
| Ti_49_-Cu_51_ | 5 × 10^6^ | 0.301$\pm$0.037 | 1 × 10^5^ | 1$\pm$0.013 | | | 10 | | 5$\pm$0.021 | | 10 | | 5$\pm$0.034 | |  |
| Ti_29_-Cu_71_ | 6.3 × 10^4^ | 1.2$\pm$0.011 | 10 | 5$\pm$0.020 | | | 10 | | 5$\pm$0.028 | | 10 | | 5$\pm$0.041 | |  |
| Ti_14_-Cu_86_ | 1 × 10^4^ | 2$\pm$0.019 | 10 | 5$\pm$0.021 | | | 10 | | 5$\pm$0.026 | | 10 | | 5$\pm$0.051 | |  |







**Figure 9S.** The FESEM images of control *E. coli* bacteria^1^


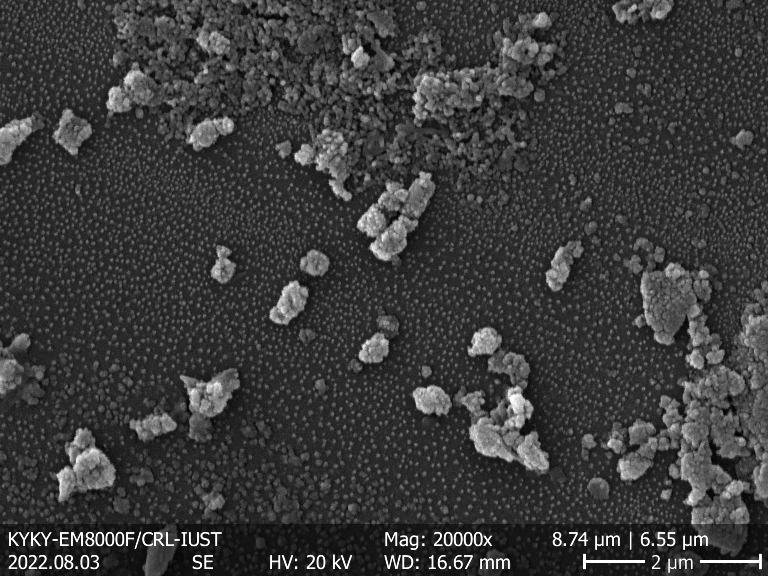




**Figure 10S.** The FESEM images of control *S. aureus* bacteria^1^


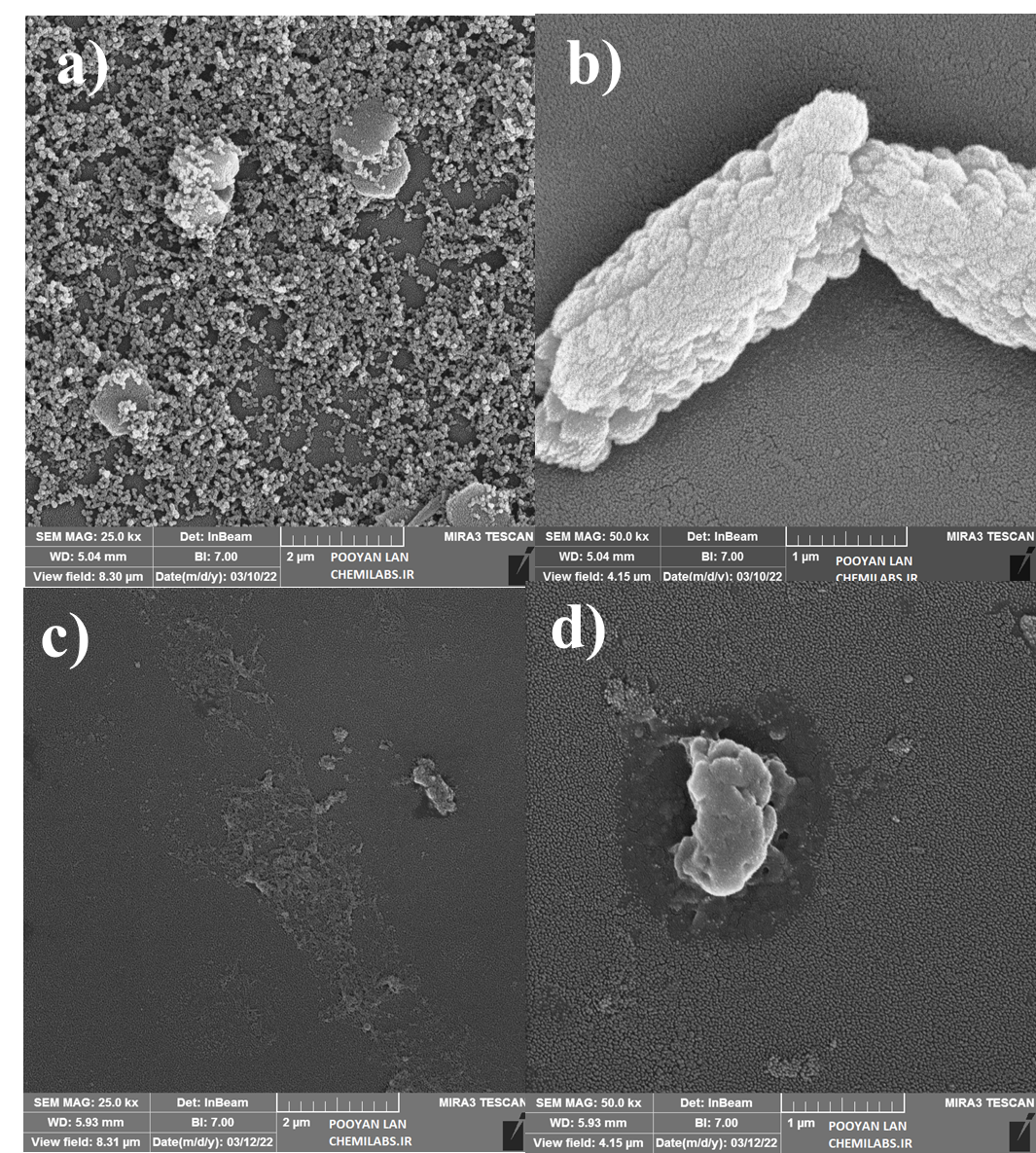


**Figure 11S.** The raw data of FESEM images of E. coli bacteria in the presence of Ti_49_-Cu_51_ thin film in scale a) 2μm b) 1μm and Ti_14_-Cu_86_ thin film in scale a) 2μm b) 1μm.


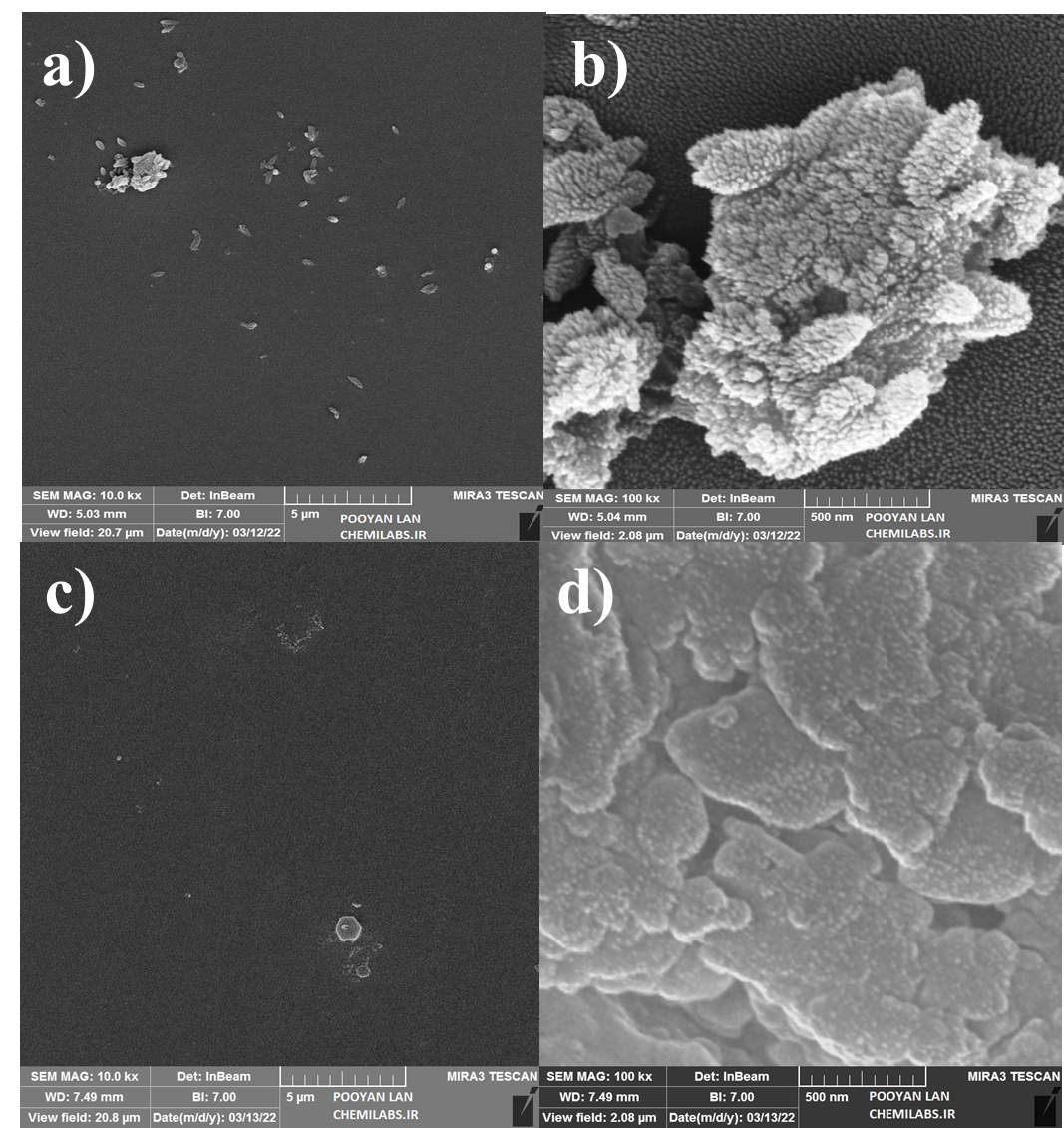


**Figure 12S.** The raw data of FESEM images of S. aureus bacteria in the presence of Ti_49_-Cu_51_ thin film in scale a) 5μm b) 500nm and Ti_14_-Cu_86_ thin film in scale a) 5μm b) 500nm.


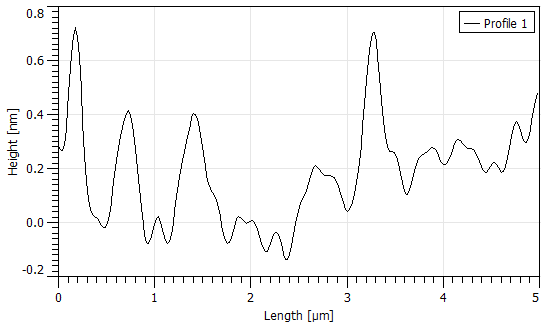


**Figure 13S.** Extracted line profile of AFM Ti thin film


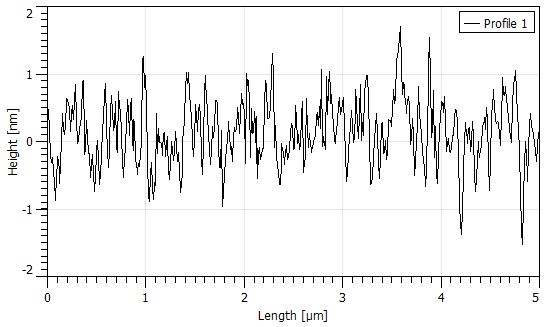


**Figure 14S.** Extracted line profile of AFM Ti_81_-Cu_19_ thin film


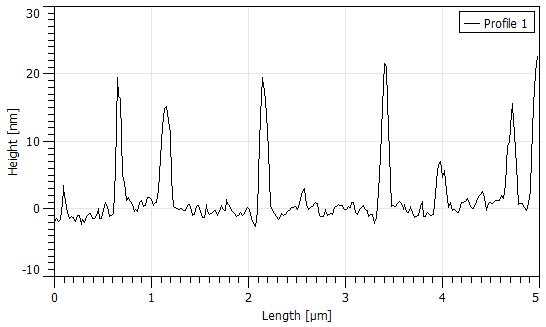


**Figure 15S.** Extracted line profile of AFM Ti_49_-Cu_51_ thin film


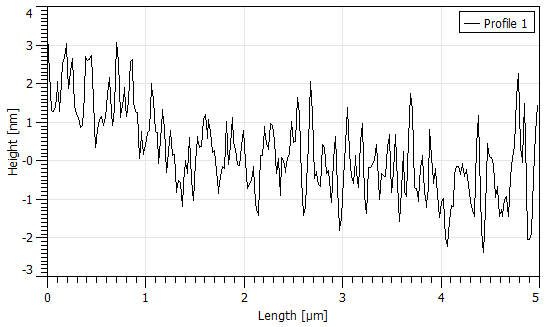


**Figure 16S.** Extracted line profile of AFM Ti_29_-Cu_71_ thin film


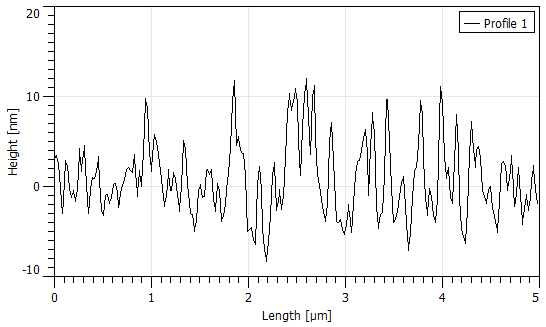


**Figure 17S.** Extracted line profile of AFM Ti_14_-Cu_86_ thin film

**References**

1. Nosrati, P., Rahimi, R. & Hosseini-Kharat, M. Investigation of antibacterial photodynamic inactivation in urea-doped TiO2 sensitized with porphyrin photocatalysis. *J. Porphyr. Phthalocyanines* **27,** 873–876 (2023).
